# Discovery of a Promising Hydroxyamino-Piperidine HDAC6 Inhibitor via Integrated Virtual Screening and Experimental Validation in Multiple Myeloma

**DOI:** 10.3390/ph18091303

**Published:** 2025-08-29

**Authors:** Federica Chiera, Antonio Curcio, Roberta Rocca, Ilenia Valentino, Massimo Gentile, Stefano Alcaro, Nicola Amodio, Anna Artese

**Affiliations:** 1 Dipartimento di Scienze della Salute, Università Magna Græcia, 88100 Catanzaro, Italy; f.chiera@unicz.it (F.C.); antonio.curcio@unicz.it (A.C.); alcaro@unicz.it (S.A.); artese@unicz.it (A.A.); 2 Associazione CRISEA-Centro di Ricerca e Servizi Avanzati per l’Innovazione Rurale, Località Condoleo di Belcastro, 88100 Catanzaro, Italy; 3 Dipartimento di Medicina Sperimentale e Clinica, Università Magna Græcia, 88100 Catanzaro, Italy; ilenia.valentino30@gmail.com; 4 Hematology Unit, Azienda Ospedaliera Annunziata, 87100 Cosenza, Italy; massimo.gentile@unical.it; 5 Department of Pharmacy, Health and Nutritional Science, University of Calabria, 87036 Rende, Italy

**Keywords:** pharmacophore, HDAC6, docking, molecular dynamics, multiple myeloma

## Abstract

**Background:** Histone deacetylase 6 (HDAC6) is a unique class IIb HDAC isozyme characterized by two catalytic domains and a zinc finger ubiquitin-binding domain. It plays critical roles in various cellular processes, including protein degradation, autophagy, immune regulation, and cytoskeletal dynamics. Due to its multifunctional nature and overexpression in several cancer types, HDAC6 has emerged as a promising therapeutic target. **Methods:** In this study, we employed a ligand-based pharmacophore modeling approach using a structurally diverse set of known HDAC6 inhibitors. This was followed by the virtual screening of over 140,000 commercially available compounds from both the MolPort and Asinex databases. The screening workflow incorporated pharmacophore filtering, molecular docking, and molecular dynamic (MD) simulations. Binding free energies were estimated using Molecular Mechanics Generalized Born Surface Area (MM-GBSA) analysis to prioritize top candidates. A fluorometric enzymatic assay was used to measure HDAC6 activity, while cell viability assay by Cell Titer Glo was used to assess the anti-tumor activity against drug-sensitive and -resistant multiple myeloma (MM) cells. Western blotting was used to evaluate the acetylation of tubulin or histone H4 after treatment with selected compounds. **Results:** Three promising compounds were identified based on stable binding conformations and favorable interactions within the HDAC6 catalytic pocket. Among them, Molecular Mechanics Generalized Born Surface Area (MM-GBSA) analysis identified **Compound 10** (AKOS030273637) as the top theoretical binder, with a ΔG_bind_ value of −45.41 kcal/mol. In vitro enzymatic assays confirmed its binding to the HDAC6 catalytic domain and inhibitory activity. Functional studies on MM cell lines, including drug-resistant variants, showed that **Compound 10** reduced cell viability. Increased acetylation of α-tubulin, a substrate of HDAC6, likely suggested on-target mechanism of action. **Conclusions: Compound 10**, featuring a benzyl 4-[4-(hydroxyamino)-4-oxobutylidene] piperidine-1-carboxylate scaffold, demonstrates potential drug-like properties and a predicted bidentate zinc ion coordination, supporting its potential as an HDAC6 inhibitor for further development in hematologic malignancies.

## 1. Introduction

Histone deacetylases (HDACs) are a group of epigenetic enzymes that regulate gene expression without altering the underlying DNA sequence. They function by removing acetyl groups from lysine residues on both histone and non-histone proteins, thereby modulating intracellular acetylation levels in concert with histone acetyltransferases [1,2]. In mammals, 18 HDAC isozymes have been identified and are classified into four groups based on their sequence similarity to yeast orthologues: Class I (HDAC1, HDAC2, HDAC3, HDAC8), Class IIa (HDAC4, HDAC5, HDAC7, HDAC9), Class IIb (HDAC6, HDAC10), Class III (sirtuins 1–7), and Class IV (HDAC11).

HDAC6 is the largest member of the HDAC family, comprising 1215 amino acids. It is characterized by two tandem catalytic domains (CD1 and CD2), located in the N-terminal and central regions, respectively [3]. Its C-terminal region contains a zinc finger ubiquitin-binding domain (ZnF-UBP), which mediates interactions with the ubiquitin-proteasome and aggresome pathways, thereby contributing to the clearance of misfolded proteins [4].

In 2016, Hai and Christianson resolved the crystal structure of HDAC6’s catalytic domains CD1 and CD2 using X-ray crystallography [5], providing crucial insights into its structure and function. Their findings showed that CD2 possesses broader substrate specificity and greater catalytic activity than CD1, primarily due to distinct structural features within their active sites. This broader specificity refers to enzymological studies showing that CD2 can act on a variety of peptide substrates, whereas CD1 exhibits much narrower specificity. The catalytic Zn^2+^ ion in CD2 plays a pivotal role in ligand recognition, with monodentate coordination being particularly favorable for specific inhibitor classes [6,7,8].

HDAC6 also contains a serine/glutamate-rich tetradecapeptide repeat domain that facilitates interactions with microtubule-associated proteins such as Tau, thereby contributing to the regulation of protein aggregation [9]. Misfolded proteins have a strong tendency to aggregate, thereby disrupting essential cellular processes, and the identification of the aggresome has significantly advanced our understanding of the cellular mechanisms involved in their recognition, processing, and degradation. Aggresomes form in response to the ectopic expression of various misfolded proteins and serve as central depots for aggregated, polyubiquitinated protein species, and HDAC6 plays a pivotal role in this pathway, being selectively involved in aggresome formation through its interaction with polyubiquitinated proteins. At the molecular level, HDAC6 simultaneously binds to ubiquitinated misfolded proteins and the motor protein dynein. This dual interaction facilitates the retrograde transport of protein aggregates along microtubules toward the microtubule-organizing center, where aggresomes form. Disruption of HDAC6 function impairs the delivery of these polyubiquitinated proteins to dynein, compromises aggresome assembly, and hampers the clearance of misfolded proteins from the cytoplasm. Consequently, cells deficient in HDAC6 exhibit heightened sensitivity to proteotoxic stress, ultimately triggering apoptotic cell death [10]. Due to its unique structural characteristics and broad substrate specificity, HDAC6 plays other multifaceted roles in various cellular processes. It regulates cytoskeletal dynamics via tubulin deacetylation, modulates cellular stress responses through the promotion of autophagy, and influences immune signaling by deacetylating key mediators, such as those involved in the NF-κB pathway, thereby affecting inflammation and immune regulation. Additionally, HDAC6 indirectly modulates gene expression by modifying non-histone proteins involved in transcriptional control [9].

These diverse functions make HDAC6 essential for maintaining cellular homeostasis and responding to stress while also establishing it as a compelling therapeutic target in diseases such as cancer, neurodegeneration, and inflammation. Recent studies have shown that HDAC6 overexpression is associated with increased cell proliferation, enhanced migratory capacity, poor clinical prognosis, and the development of drug resistance across various cancer types [11,12]. Given its distinctive structural and functional roles, HDAC6 has emerged as a promising target for therapeutic intervention. Selective inhibition of HDAC6 has demonstrated notable potential in attenuating tumor progression, reducing metastasis, and overcoming drug resistance in a range of preclinical cancer models [13,14].

Currently approved HDAC inhibitors (HDACis), such as vorinostat and panobinostat, exhibit limited isozyme selectivity, often inhibiting both Class I and Class II HDACs. This broad-spectrum activity affects multiple cellular pathways and is associated with significant adverse effects [15,16]. Consequently, there is growing interest in developing selective HDAC6 inhibitors. Several candidates are currently under preclinical and clinical investigation. Notably, some hydroxamic acid-based compounds have demonstrated high selectivity for HDAC6 and the ability to cross the blood–brain barrier, exhibiting neuroprotective effects in various disease models [17].

Furthermore, Lv et al. reported a new class of brain-penetrant, non-hydroxamate HDAC6-selective inhibitors designed to minimize the genotoxicity commonly associated with hydroxamate-based scaffolds [18].

Although several pan-HDAC inhibitors have been approved by the FDA, they are currently restricted mainly to the treatment of cutaneous T-cell lymphoma [19]. More recent preclinical research has focused on the potential of HDAC6 inhibitors, particularly in combination therapies for cancers such as melanoma, glioblastoma, and various hematological malignancies [20,21,22,23], including multiple myeloma (MM), a still incurable plasma cell disorder characterized by abnormal proliferation of malignant plasma cells in the bone marrow [24,25].

Multiple myeloma (MM) represents the second most common hematologic malignancy worldwide and is defined by the clonal expansion of neoplastic plasma cells within the bone marrow microenvironment. Its pathogenesis involves a multifactorial process driven by intricate genetic and epigenetic alterations [24,26,27], including chromosomal translocations, copy number variations, and dysregulation of critical signaling pathways [28,29,30], which collectively promote cellular proliferation, survival, and resistance to apoptosis.

Despite substantial therapeutic advancements—such as the incorporation of proteasome inhibitors, immunomodulatory agents, monoclonal antibodies [31], immune-based therapies, and a growing array of emerging targeted agents—MM remains an incurable malignancy for the majority of patients. Most individuals ultimately experience disease relapse and progression, frequently accompanied by increasing resistance to available therapeutic modalities [32,33,34,35,36]. Moreover, complications such as peripheral neuropathy can significantly impact patients’ quality of life and limit therapeutic options [37].

In this regard, HDAC6 inhibitors have demonstrated encouraging anti-MM activity, mainly due to their capability to disrupt the aggresome-autophagy pathway and induce apoptosis in MM cells. This pro-apoptotic effect increases when using these inhibitors in combination with proteasome inhibitors. In addition to their cytotoxic properties, HDAC6 inhibitors enhance antitumor immune responses, contributing further to their therapeutic potential in MM [38]. According to ClinicalTrials.gov, two HDAC6 inhibitors (**ACY-1215** and ACY-241) have advanced into clinical trials for MM therapy. Despite their low nanomolar half-maximal inhibitory concentration (IC_50_) values against HDAC6, both compounds exhibit only modest selectivity, with approximately 10-fold higher affinity for HDAC6 over class I isozymes HDAC1, HDAC2, and HDAC3 [39]. Given these limitations, and the well-established role of HDAC6 in myelomagenesis, recent research efforts have been directed toward the development of next-generation HDAC6 inhibitors with enhanced potency and selectivity. Notably, a novel irreversible inhibitor incorporating a phenylsulfonylfuroxan-based hydroxamate scaffold has shown improved anti-MM activity [38], underscoring ongoing advancements in this therapeutic area.

Based on these insights, we performed a Ligand-Based Pharmacophore Screening (LBPS) of commercially available compound libraries, using a structurally diverse set of known HDAC6 inhibitors with established anticancer activity. Subsequent molecular docking and molecular dynamic simulations led to the identification of three promising candidates as potential HDAC6 inhibitors. Among them, AKOS030273637 (**Compound 10**), featuring a benzyl 4-[4-(hydroxyamino)-4-oxobutylidene]piperidine-1-carboxylate scaffold, was confirmed to inhibit HDAC6 enzymatic activity, with a significant anti-tumor effect against MM cell lines, including those resistant to standard therapies.

## 2. Results

### 2.1. Identification of Potential HDAC6 Inhibitors via Virtual Screening

In this study, the computational pipeline was launched by downloading and preparing the HDAC6 isozyme structure (PDB ID: 5EDU) [40], along with compound libraries from both MolPort Natural Compounds [41] and Asinex (BioDesign Library and Elite Library) [42], which comprise a total of over 300,000 molecules. To minimize stereochemical ambiguity and circumvent issues related to enantiomeric purity, the database was filtered based on molecular chirality, retaining only achiral compounds. This step refined the screening library to 142,486 entries. Subsequently, a ligand-based pharmacophore model was generated using a *training set* of potent HDAC6 inhibitors with different structural motifs (Table 1 and Table S1).

**Figure 1:**
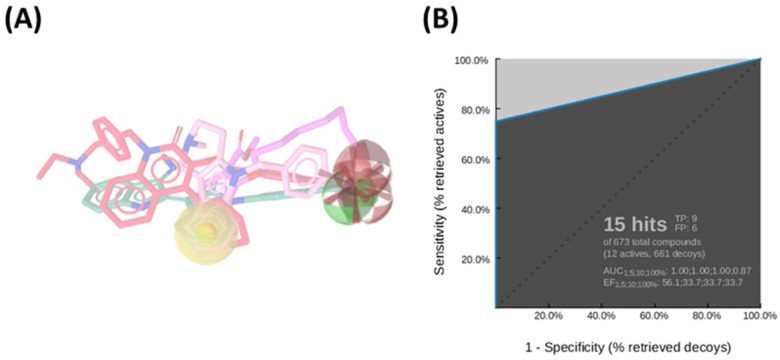
(**A**) Ligand-based pharmacophore model derived from a diverse set of known HDAC6 inhibitors, highlighting key pharmacophoric features: hydrogen bond donors (green spheres), hydrogen bond acceptors (red spheres), hydrophobic regions (yellow sphere), and ionizable groups (red arrows). (**B**) Receiver Operating Characteristic (ROC) curve generated by automated analysis in LigandScout [43], demonstrating the model’s ability to discriminate between active and inactive compounds.

Among the ten generated pharmacophore hypotheses (Table S2), the top-ranked model—featuring two hydrogen bond acceptors (HBA), one hydrogen bond donor (HBD), one hydrophobic feature (NI), and one negatively ionizable group (HY)—exhibited strong predictive performance, achieving an area under the curve (AUC) of 87.0% and a sensitivity of 75% in virtual screening assays (Figure 1A). This model served as an initial screening tool to prioritize compounds for subsequent structure-based analysis.

More in detail, the final pharmacophore model is defined by two HBAs and one HBD, all spatially localized within the hydroxamic acid moiety, a functional group conserved across all training compounds and known to play a pivotal role in zinc coordination within HDAC active sites. A HY feature is also present and corresponds to different aromatic systems that contribute to ligand-enzyme interactions via π–π stacking and hydrophobic contacts. These include: the phenyl ring of the dihydroquinoline scaffold in **Compound 2**, the phenyl moiety of the N-phenylaniline group in **Compound 6**, the naphthalene ring system in **Compound 1**, the phenyl ring fused to the indole core in **Compound 3**, the pyridine ring in **Compound 4**, and the pendant phenyl substituent on the indole system in **Compound 5** (Figure S1).

**Figure 2:**
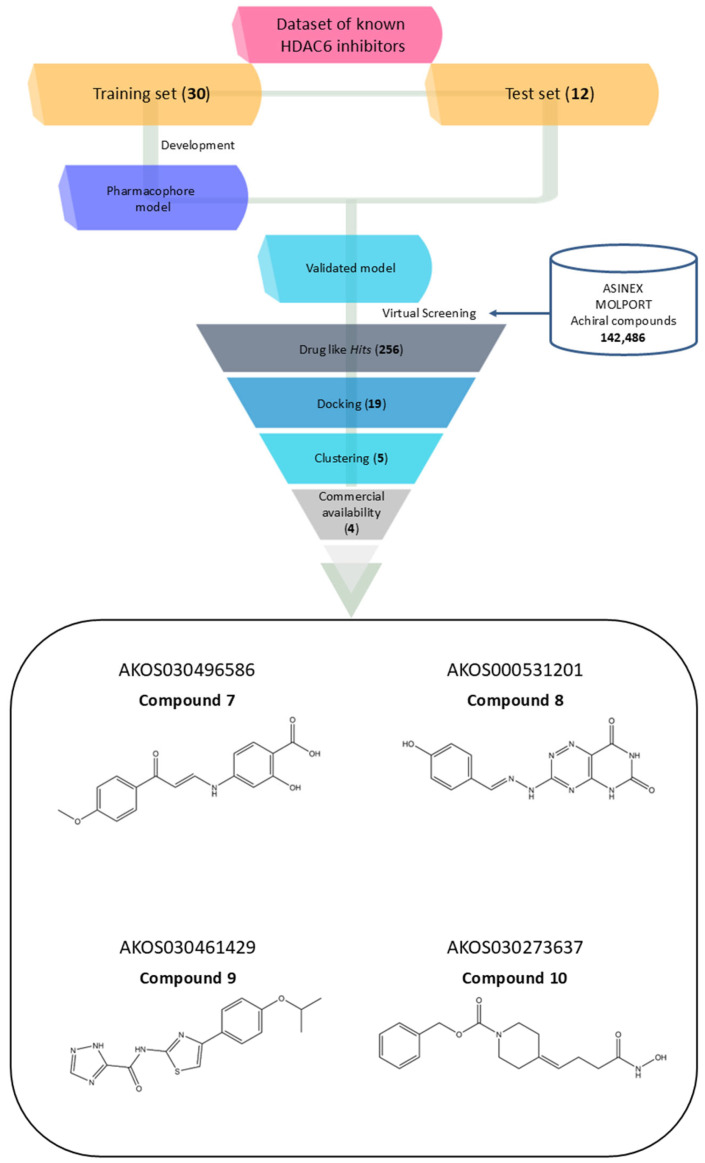
Workflow of the virtual screening process for discovering novel HDAC6 inhibitors, accompanied by the 2D structures of four selected compounds prioritized for experimental validation.

Notably, the model does not incorporate a defined linker region, which is typically observed in classical HDAC inhibitor pharmacophores bridging the cap group and the zinc-binding domain. This absence could be attributed to the structural heterogeneity of the training set, prompting the generation of a consensus pharmacophore based solely on features that are universally conserved across all compounds. Pharmacophore validation was conducted through the virtual screening of the curated test set libraries, followed by receiver operating characteristic (ROC) curve analysis. The model demonstrated high discriminative power, correctly identifying 9 out of 12 active compounds, with a sensitivity of approximately 75% and an area under the ROC curve (AUC) of 0.87 (Figure 1B). This validated pharmacophore model was subsequently utilized to filter the prepared compound libraries, prioritizing candidates based on optimal pharmacophore fit scores. This refinement step yielded a subset of 256 compounds—comprising 101 from MolPort, 26 from Asinex BioDesign Library, and 129 from Asinex Elite Library. These selected *hits* were then subjected to a structure-based virtual screening (SBVS) workflow to further assess their binding potential within the HDAC6 catalytic site (Figure 2).

To establish a reliable threshold for the subsequent SBVS, we performed re-docking of the co-crystallized ligand **Trichostatin A** (TSA) into both chain A and chain B of the prepared HDAC6 model. Chain A was selected for further docking studies as its re-docked pose yielded the lowest root-mean-square deviation (RMSD) of 1.12 Å, confirming the accuracy and reproducibility of the docking protocol. The Glide Score (G-Score) of **Trichostatin A**’s best pose in chain A was used as a cut-off criterion. A total of 19 compounds demonstrated better G-Scores than **Trichostatin A**.

These 19 *hits* were subjected to fingerprint-based clustering using the Tanimoto coefficient. The optimal number of clusters was determined with Canvas software (version 3.9) [44] employing the Kelley criterion, resulting in five distinct clusters. For each cluster, the representative compound was identified by selecting the molecule with the highest number of shared features relative to other cluster members, based on centroid comparison (Figure S2). Among the five identified molecules, only four were commercially available from the same supplier. As a result, AKOS030314251 was excluded, and the remaining four were selected for further analysis (Figure 2, Table 2).

The four selected *hit* compounds exhibit distinct chemical scaffolds and incorporate structural features consistent with HDAC6 inhibition. **Compound 7** (AKOS030496586) integrates phenolic, carboxylic, and enone functionalities, allowing for a combination of polar interactions and extended π-conjugation within the active site. The second *hit*, **Compound 8** or AKOS000531201, possesses a fused heterocyclic system capable of forming multiple hydrogen bonds and π–π stacking interactions, along with a methoxy-substituted aromatic ring likely to engage hydrophobic regions of the binding pocket. **Compound 9** (AKOS030461429) features a thiazole-triazole core and a hydrophobic isopropoxyphenyl moiety, suggesting potential for both metal coordination and hydrophobic interactions within the HDAC6 active site. Finally, **Compound 10** (AKOS030273637) contains a hydroxamic acid group—a well-established zinc-binding motif in HDAC inhibitors—linked to a piperidine scaffold, which may contribute to improved binding specificity and physicochemical properties.

**Figure 3:**
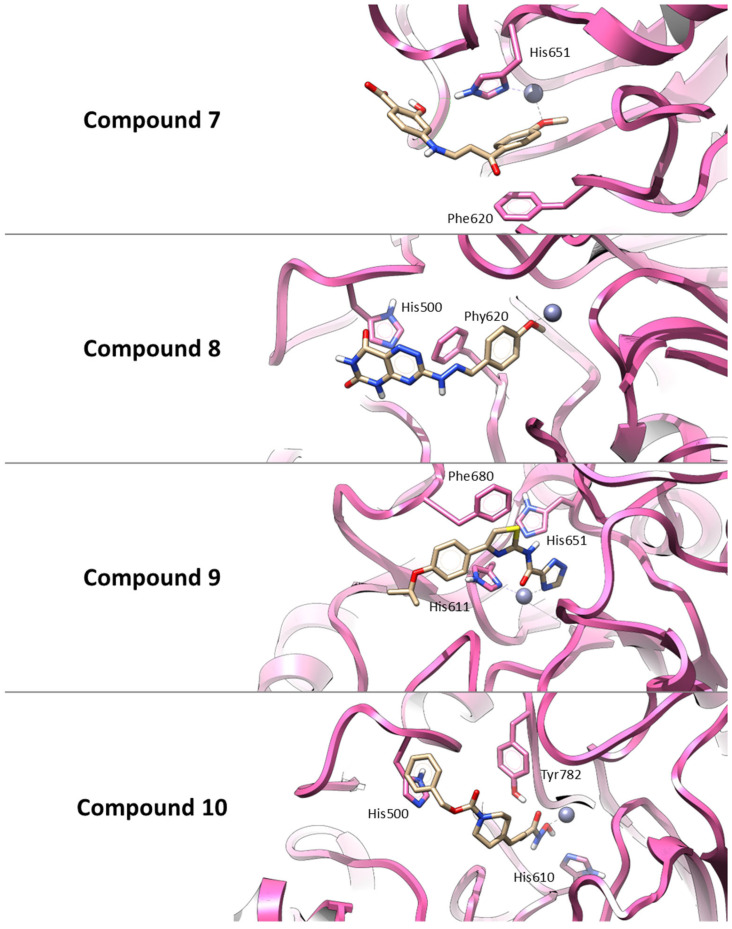
Three-dimensional representations of the best docking poses for the four *hit* compounds selected from the virtual screening. The binding conformations within the HDAC6 active site are shown for: **Compound 7** (AKOS030496586), **Compound 8** (AKOS000531201), **Compound 9** (AKOS030461429) and **Compound 10** (AKOS030273637). Ligands are represented as yellow carbon sticks, the protein is depicted as a mauve ribbon, and the catalytic zinc ion is shown as a gray sphere. Key interacting residues are highlighted to illustrate binding interactions.

Thus, similar to **Trichostatin A**, all selected compounds possess functional groups capable of coordinating the catalytic zinc ion within the HDAC6 active site. Also, **Compound 7** establishes a π–π interaction with Phe620 and a hydrogen bond with His651, supporting a combination of polar and aromatic interactions. **Compound 8** primarily interacts through π–π stacking with His500 and Phe620, indicating a predominantly hydrophobic binding profile. **Compound 9** is predicted to engage in three π–π stacking interactions with His611, His651, and Phe680, stabilizing its binding conformation. Finally, **Compound 10** forms hydrogen bonds with His610 and Tyr782, along with a π–π interaction involving His500, suggesting a strong and multifaceted binding mode. These interaction patterns are illustrated in Figure 3.

To gain deeper insights into the molecular recognition mechanisms of HDAC6, molecular dynamic simulations (MDs) were conducted in explicit water on complexes of the four selected *hit* compounds. As a control, molecular dynamics (MD) simulations were also performed on **Trichostatin A** to validate the simulation protocol. TSA is a well-characterized broad-spectrum (pan-HDAC) inhibitor, exhibiting markedly higher potency against class I HDAC isoforms, such as HDAC1, than against class IIb enzymes like HDAC6.

The RMSD plots of the heavy atoms of the protein for all protein–ligand complexes, including **Trichostatin A** and the selected *hit* compounds, are shown in Figure S3. This figure also includes a table summarizing the average RMSD values for each system, providing a clear overview of the protein’s structural stability throughout the simulations.

**Figure 4:**
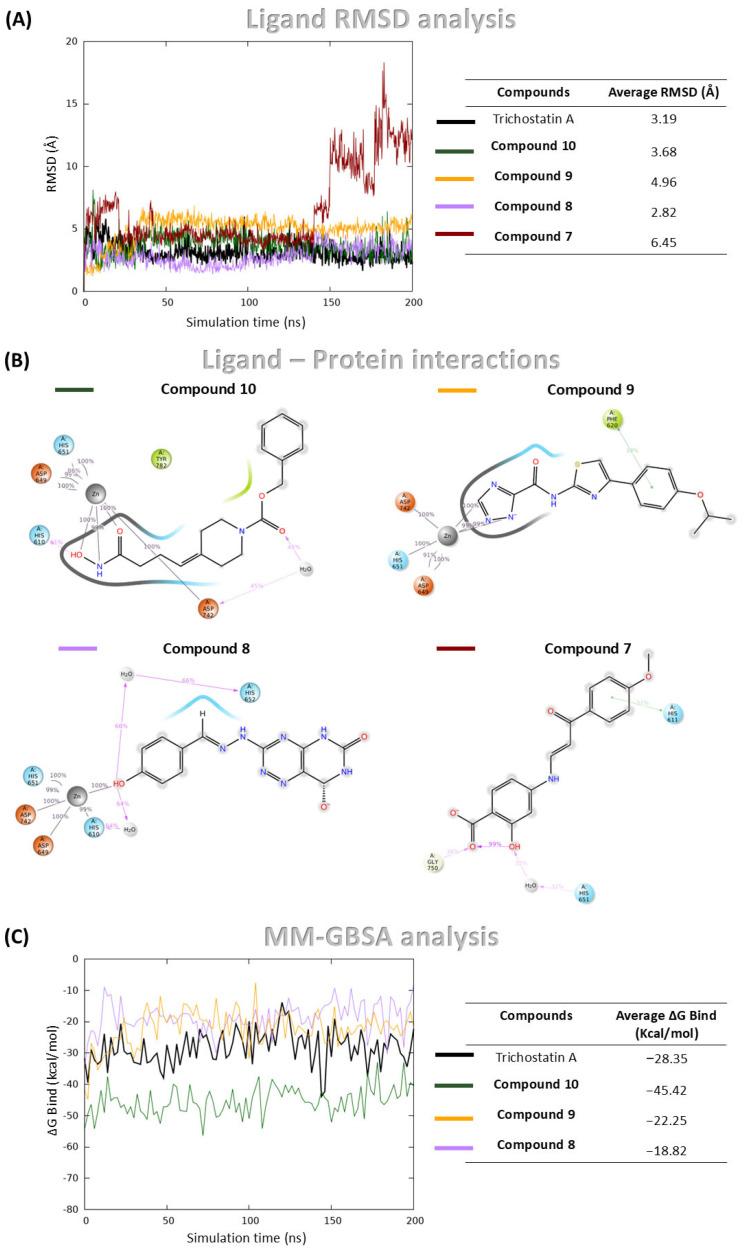
MDs and MM-GBSA analysis of **Compound 8**, **Compound 10**, **Compound 9**, **Compound 7**, and **Trichostatin A** (used as control) in complex with HDAC6. (**A**) RMSD of ligand heavy atoms over the simulation, expressed in Å; (**B**) Protein-ligand interaction profile showing contacts between ligand atoms and HDAC6 residues. Only interactions occurring for more than 30% of the 200 ns simulation time are displayed; (**C**) Binding free energy profiles calculated using the MM-GBSA method throughout the simulation.

**Trichostatin A** demonstrated stable binding throughout the simulation, as indicated by consistently low RMSD values of ligand heavy atoms, calculated by aligning trajectory frames to the protein backbone of the initial structure (Figure 4A). Additionally, it maintained coordination with the catalytic zinc ion over the full simulation time (Figure S4), confirming the robustness of the simulation setup.

Among the four *hit* compounds, **Compound 8**, **Compound 9** and **Compound 10** exhibited stable binding profiles, maintaining low RMSD values and sustained interactions with the catalytic zinc ion (Figure 4A,B). In contrast, **Compound 7** initially remained bound, but during the final 50 ns of the simulation, it dissociated from the active site and lost zinc coordination (Figure 4A,B).

Detailed interaction analyses revealed that **Compound 8** formed a stable π–π interaction with Phe620, **Compound 10** was involved in a hydrogen bond with His610 and a water bridge with Asp742, and **Compound 9** established two water bridges with His610 and His652 (Figure 4B).

To complement the structural stability analysis, Molecular Mechanics Generalized Born Surface Area (MM-GBSA) calculations were performed in order to estimate binding free energies (ΔG_bind_) for the three stably bound compounds and **Trichostatin A**. While MM-GBSA offers a valuable approximation of binding affinity, it predominantly reflects enthalpic contributions and does not explicitly account for entropic factors, which can also significantly influence ligand binding. **Trichostatin A** displayed a mean ΔG_bind_ of −28.35 kcal/mol. Notably, **Compound 10** exhibited a more favorable binding energy of −45.41 kcal/mol, while **Compounds 9** and **8** showed values of −18.82 kcal/mol and −22.25 kcal/mol, respectively (Figure 4C).

Based on these results, all three compounds were selected for purchase and subjected to subsequent experimental validation.

### 2.2. Fluorometric Validation of HDAC6 Inhibition: ***Compound 10*** Emerges as a Potent Lead Compound

The ability of **Compound 8**, **Compound 9**, and **Compound 10** to inhibit HDAC6 enzymatic activity was assessed using a fluorometric assay designed to measure HDAC6-specific deacetylation activity. The results, summarized in Table 3, demonstrate that **Compound 10** was the most potent among the three compounds, exhibiting an IC_50_ in the low micromolar range.

Notably, the IC_50_ value of **Compound 10** was significantly lower compared to those of **Compound 8** and **Compound 9**, indicating a stronger inhibitory effect on HDAC6 (Table 3; Figure S5). These findings are consistent with prior in silico predictions and support the selection of **Compound 10** as the lead compound for further biological validation and mechanistic studies.

### 2.3. ***Compound 10*** Inhibits Multiple Myeloma (MM) Cell Viability Enhancing Acetylated α-Tubulin Expression

**Figure 5:**
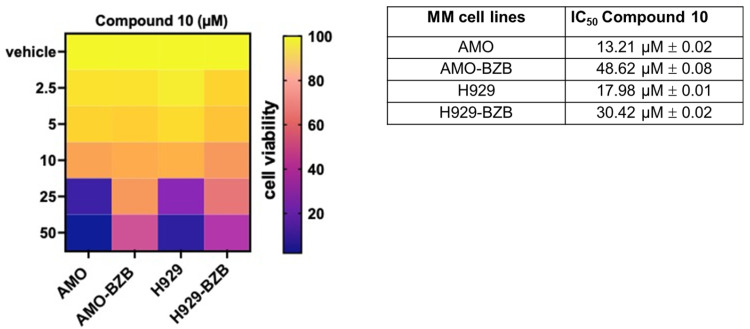
Heatmap showing cell viability assessed by the CellTiter-Glo assay in MM cell lines treated with **Compound 10** or vehicle control (DMSO) for 72 h. Viability is expressed as a percentage relative to vehicle-treated cells. The half-maximal inhibitory concentrations (IC_50_) of **Compound 10** for AMO, AMO-BZB, H929, and H929-BZB cell lines are reported in the accompanying table. IC_50_ values (mean ± SD) were calculated using GraphPad Prism software based on three independent experiments.

Next, we investigated the in vitro anti-tumor activity of **Compound 10** in MM cell lines, including those that are either sensitive or resistant to standard-of-care treatments, such as proteasome inhibitors (PIs). Notably, treatment with **Compound 10** resulted in a significant reduction in cell viability across all tested MM cell lines, exhibiting half-maximal inhibitory concentration (IC_50_) values in the micromolar range, thereby demonstrating potent cytotoxic effects. Importantly, the IC_50_ values were approximately two-fold lower in PI-sensitive cell lines compared to PI-resistant counterparts (Figure 5), suggesting that **Compound 10** is more effective in treatment-naïve or less refractory MM contexts.

Compared to the reference HDAC6-selective compound ACY1215, **Compound 10** exhibited higher IC_50_; moreover, similar to **Compound 10**, **ACY-1215** also displayed greater activity in PI-sensitive cell lines compared to their resistant counterparts (Figure S6). Interestingly, **Compound 10** elicited negligible effects on the viability of non-transformed cells (293T) or healthy peripheral blood mononuclear cells (PBMCs), suggesting a favorable therapeutic window (Figure S7).

**Figure 6:**
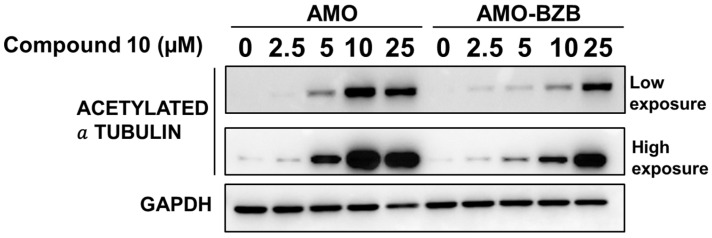
Western blot analysis of acetylated α-tubulin in AMO and AMO-BZB cells 72 h after treatment with **Compound 10**. GAPDH was used as a loading control.

We next evaluated whether HDAC6 inhibition mediated by **Compound 10** could translate into increased acetylation of known HDAC6 substrates, such as α-tubulin. Similarly to the HDAC6-selective compound **ACY-1215** (Figure S8), treatment with **Compound 10** led to a marked increase in acetylated α-tubulin levels in MM cell lines, including both drug-sensitive and drug-resistant models (Figure 6).

This increase in tubulin acetylation provides a functional readout of HDAC6 inhibition and confirms that **Compound 10** effectively engages its target in a cellular context. Notably, the effect was observed regardless of the cells’ sensitivity to proteasome inhibitors, indicating that **Compound 10** can likely modulate HDAC6 activity across diverse MM phenotypes.

In comparison to its effect on acetylated α-tubulin, the impact on histone H4 acetylation was moderate and even lower than that induced by **ACY-1215**, further supporting the HDAC6-inhibitory activity of this compound (Figure S9).

## 3. Discussion

HDAC6 is a unique member of the histone deacetylase family, distinguished by its structural features, including two catalytic domains and a zinc finger ubiquitin-binding domain, as well as its broad substrate specificity. HDAC6 is therefore involved in a variety of cellular processes, such as protein degradation, cell motility, stress responses, and autophagy. Consequently, it plays a pivotal role in maintaining cellular homeostasis and modulating the response to diverse forms of stress. Its involvement in multiple oncogenic and neurodegenerative pathways has positioned HDAC6 as a highly promising therapeutic target in conditions such as cancer, neurodegeneration, and inflammation.

In recent years, the selective inhibition of HDAC6 has garnered considerable interest as a therapeutic strategy to attenuate tumor progression, suppress metastasis, and overcome drug resistance—particularly in preclinical models of cancer. Given its therapeutic potential, significant efforts have been directed toward the identification of novel, selective inhibitors. Among these, pharmacophore-based virtual screening has emerged as a valuable approach for discovering new scaffolds with preferential affinity for HDAC6 [45,46,47,48].

Most HDAC inhibitors (HDACis), including those targeting HDAC6, incorporate a hydroxamate moiety as the zinc-binding group (ZBG), which chelates the catalytic Zn^2+^ ion within the enzyme’s active site [49,50]. However, because this functional group is common to inhibitors of various zinc-dependent HDAC isozymes, structural selectivity must be conferred through modifications to other regions of the molecule—namely, the linker and capping groups.

In our computational drug discovery workflow, we employed a multitiered in silico approach, combining ligand-based pharmacophore modeling, molecular docking, molecular dynamic simulations, and MM-GBSA binding free energy calculations. For this initial proof-of-concept study, we deliberately focused on hydroxamate-based inhibitors, given their high prevalence in public datasets and well-characterized zinc-chelating interactions with HDAC active sites. This chemically homogeneous subset enabled the generation of a consistent and reliable pharmacophore model, leveraging the conserved binding mode of hydroxamic acids to produce a robust and predictive hypothesis.

We acknowledge that this focus represents a limitation in terms of chemical diversity. While including alternative zinc-binding groups (ZBGs) could enhance the model’s generalizability, their incorporation would require a significantly larger and more balanced training set to maintain model accuracy and avoid introducing structural noise. Nevertheless, the present model provides a strong foundation that can be systematically expanded in future studies to accommodate a broader range of ZBGs within a more comprehensive virtual screening strategy.

This approach led to the identification of three promising candidate compounds. Among them, **Compound 10** exhibited the most favorable binding energy profile, outperforming the reference inhibitor **Trichostatin A**. This compound, abenzyl 4-[4-(hydroxyamino)-4-oxobutylidene]piperidine-1-carboxylate, was sourced from the Asinex BioDesign Library and has a molecular weight of 318.37 g/mol. According to SwissADME predictions, it displays favorable drug-likeness and medicinal chemistry properties [51]. Structurally, it conforms to the classical HDAC inhibitor architecture, comprising a ZBG, linker, and capping group. Importantly, it avoids the use of bulky capping moieties, which are often associated with reduced ligand efficiency and off-target activity. Interestingly, structurally related compounds have previously been identified as HDAC inhibitors. In detail, various alkyl piperidine hydroxamic acids exhibited submicromolar inhibitory activity. Among these, the urea analogs were the most potent, although sulfonamides and amides were also investigated [52,53]. **Compound 10** differs from this series of alkyl piperidine hydroxamic acids by the presence of an unsaturation within the aliphatic chain, which reduces molecular flexibility. Moreover, it features a carbamate moiety instead of the urea, sulfonamide, or amide groups previously described.

Of particular note is its ability to form a stable hydrogen bond with His610, a residue located near the catalytic zinc ion. This interaction resembles the dual coordination mechanism observed in TO-317, a highly selective HDAC6 inhibitor that engages both Zn^2+^ and His614 in *Danio rerio* HDAC6 [54]. While these findings suggest a potentially favorable interaction pattern for HDAC6 engagement, it is important to emphasize that isozyme selectivity was not explicitly assessed in our computational protocol and has not yet been validated experimentally. Therefore, further studies are required to evaluate the selectivity profile and biological activity of these compounds across different HDAC isozymes.

After confirming the ability of **Compound 10** to enzymatically inhibit HDAC6, we exploited MM as a model to investigate its anti-tumor activity. It is well established that HDAC6 plays a critical role in MM pathogenesis by regulating protein homeostasis and aggresome formation, thereby enabling MM cells to survive proteotoxic stress, particularly under proteasome inhibition [55]. HDAC6 inhibitors have demonstrated promising anti-MM activity by disrupting aggresome-mediated protein degradation and enhancing the cytotoxic effects of PIs, thereby offering a potential strategy to overcome drug resistance in MM [20,56], supporting the therapeutic relevance of HDAC6 targeting in this cancer.

Of note, **Compound 10** was able to inhibit MM cell viability in a mid-high micromolar range, with a lower IC_50_ observed in PI-sensitive cells. This differential sensitivity may reflect distinct vulnerabilities in the HDAC6 pathway between these subtypes and supports the potential use of **Compound 10** either as a monotherapy or in combination with PIs to overcome drug resistance. These findings underscore the therapeutic potential of **Compound 10** in MM and warrant further mechanistic and combinatorial studies to evaluate its efficacy in overcoming resistance to existing therapies.

Supporting its potential on-target mechanism of action, **Compound 10** treatment increased acetylation of the HDAC6 substrate α-tubulin in treated MM cell lines, while effects on acetylated histone H4 was significantly less pronounced.

Although these findings are promising, the selectivity profile of **Compound 10** against other HDAC isoforms and unrelated enzymes remains to be fully established. We acknowledge this as a limitation of the current study and consider it an important objective for follow up investigations.

However, taken together, our data demonstrate that the anti-tumor effects of **Compound 10** are closely associated with its ability to inhibit HDAC6 enzymatic activity, resulting in enhanced acetylation of downstream substrates. Finally, we can highlight the therapeutic relevance of targeting HDAC6 in MM and propose **Compound 10** as a promising candidate for further development.

## 4. Materials and Methods

### 4.1. Pharmacophore Model Generation

A set of thirty HDAC6 inhibitors (Table S1) was selected for ligand-based pharmacophore modeling based on their potent antitumor activity, defined by IC_50_ values below 100 nM and their demonstrated selectivity toward HDAC6 across various cancer models. Particular emphasis was placed on hydroxamate-based structures, given their prevalence in publicly available datasets and their well-characterized zinc-binding modes within HDAC active sites. While the hydroxamic acid moiety is a common pharmacophoric feature across HDAC inhibitors, HDAC6 selectivity is primarily influenced by the structural variability in the surface recognition domain or capping group (CAP). Therefore, the training set was carefully curated to include a diverse array of CAP structures, ensuring that this key element of selectivity was well-represented in the pharmacophore hypothesis. Ligand-based pharmacophore modeling was carried out using this training set, and the resulting hypothesis was validated with a test set comprising both active and inactive compounds to evaluate its predictive accuracy and discriminative power [43,57].

Initially, the set of thirty compounds was clustered based on chemical similarity using the Tanimoto coefficient. The optimal number of clusters was determined with the Canvas tool [44], employing the Kelly criterion as the selection strategy, which resulted in six distinct groups. The centroid molecule from each cluster, representing six structurally diverse scaffolds, was selected as the ‘training set’ for pharmacophore model generation (Table 1). These representative compounds were then used to derive a shared pharmacophore hypothesis within the Ligand-Based Modeling Perspective of LigandScout (version 4.4.6) [43]. All 2D molecular structures were initially constructed using the Maestro graphical interface of the Schrödinger Suite [58] and subsequently converted to the LBD format via the “Idbgen” utility in LigandScout. Conformer generation was performed using the OMEGA software v 3.1.1.2 (best settings), allowing for up to 200 conformations per molecule. LigandScout then derived common pharmacophore features by aligning the conformers and interpolating shared interactions based on a predefined number of optimal alignment solutions. A total of ten pharmacophore hypotheses were generated within a feature tolerance threshold of 1.0 Å and ranked using combined scoring metrics based on pharmacophore fit and atom overlap (Table S2).

Accordingly, the generated pharmacophore model was retrospectively validated to assess its effectiveness in a virtual screening (VS) context. For this purpose, two distinct libraries comprising active, inactive compounds and decoys were assembled (Tables S3–S5). Specifically, 12 known HDAC6 active compounds (Table S3) were used to generate 375 property-matched decoys (at a 1:58 ratio) via the DUD-E database (Table S4) [59]. These were combined with an additional set of 316 inactive compounds (Table S5) retrieved from ChEMBL [60], resulting in a validation set of 703 molecules (Tables S2–S4). The virtual screening of this dataset, followed by receiver operating characteristic (ROC) curve analysis, was employed to evaluate the model’s discriminatory capability.

### 4.2. Protein and Database Preparation and Docking Simulation

In our SBVS study, we carefully selected and downloaded the 3D coordinates of HDAC isozyme 6 from the Protein Data Bank (PDB) [61]. In particular, we used the crystallographic structures with the following PDB code: 5EDU [40]. We selected the protein structure based on three factors: (1) high resolution, (2) absence of mutations and (3) presence of co-crystallized inhibitor, as it provides valuable information about ligand binding and protein’s function.

The structures were prepared and energy-optimized through the Protein Preparation Wizard [62] tool implemented in Maestro [58], using OPLS_2005 as force field [63]. Thus, residual crystallographic buffer components and water molecules were removed, hydrogen atoms were added, and side chains’ protonation states were assigned at pH 7.4. The protein used consists of two chains, chain A and chain B, both complexed with **Trichostatin A**.

The docking grid was defined automatically based on the binding site of the co-crystallized ligand, **Trichostatin A**, which is present in both protein chains. Glide SP docking was performed using default settings to generate ten poses per ligand [64]. Re-docking of the reference ligand resulted in a lower root-mean-square deviation for chain A (1.12 Å) compared to chain B (5.67 Å), indicating more accurate pose reproduction and thus supporting the selection of chain A for downstream virtual screening (Figure S10). To filter potential *hits*, the Glide Score (G-Score) of the best redocked pose of **Trichostatin A** (–5.52 kcal/mol) was used as the threshold.

### 4.3. Virtual Screening Strategy and Workflow

The virtual screening was conducted against commercial compound libraries from Asinex [42] and MolPort [41], collectively comprising over 300,000 molecules, including the Asinex BioDesign and Elite Libraries. The libraries were initially prepared using the LigPrep tool [65], where hydrogens were added, salts removed, and ionization states predicted at pH 7.4 using Epik. Each structure was energy-minimized using OPLS_2005 as a force field [63]. To avoid stereochemical ambiguities and complications from enantiomers, all chiral molecules were excluded, retaining only achiral compounds for screening.

Ligand-Based Virtual Screening (LBVS) was performed using LigandScout’s Screening Perspective with a validated pharmacophore model to identify compounds matching the predefined features. The resulting *hits* were further docked using the Glide SP protocol with default settings, generating up to ten poses per ligand. Compounds with Glide G-Scores better than the established HDAC6 threshold were selected for subsequent analysis. Fingerprint clustering based on the Tanimoto coefficient was then performed to group structurally similar compounds. Using the Kelley criterion, 4 optimal clusters were identified [44]. From each cluster, the molecule closest to the centroid—exhibiting the most shared features—was chosen as the representative *hit*.

### 4.4. Molecular Dynamic Simulations and MM-GBSA Analysis

The complexes of the four selected molecules with HDAC6 were subjected to 200 ns molecular dynamic simulations using Desmond version 4.4 [66], employing OPLS_2005 as a force field [63]. As a positive control, **Trichostatin A** complexed with the Class IIb HDAC6 isozyme was also simulated. Each system was placed within an orthorhombic simulation box with a 10 Å buffer and solvated explicitly using TIP4P as a water model [67]. Counterions were added to neutralize the overall system charge.

The reversible reference system propagation algorithm (r-RESPA) was applied with multiple time steps: 2 fs for bonded and short-range non-bonded interactions and 6 fs for long-range non-bonded interactions. For short-range Coulombic interactions, a time step of 1 fs and a 9.0 Å cutoff were used. Long-range electrostatics were treated with the Smooth Particle Mesh Ewald (PME) method [68].

Following solvation, energy minimization and equilibration were carried out using the Martyna–Tobias–Klein (MTK_NPT) ensemble. The systems were equilibrated under NVT and NPT ensembles at 1 atm pressure and 300 K temperature, controlled via the Berendsen thermostat and barostat. Trajectory frames were recorded every 20 ps and analyzed using the Simulation Interaction Diagram (SID) tool to assess geometric and thermodynamic properties of the complexes.

Finally, binding free energies (ΔG_bind_) were estimated through Molecular Mechanics–Generalized Born Surface Area calculations [69], performed every 10 frames to evaluate the thermodynamic stability of the complexes.

### 4.5. Cell Cultures and Drugs

The MM cell lines used in this study were: AMO, AMO-BZB, NCI-H929 and NCI-H929-BZB. AMO and AMO-BZB cells were provided by Dr. C. Driessen (University of Tubingen, Tübingen, Germany). The NCI-H929 cell line was purchased from DSMZ (Braunschweig, Germany). Bortezomib (BZB)-resistant NCI-H929 cells were selected by incubation with increasing concentrations of the drug. Human MM cell lines were cultured in RPMI-1640 medium (Corning, Turin, Italy) supplemented with 10% of heat-inactivated fetal bovine serum (FBS) (Gibco^®^, Life Technologies, Carlsbad, CA, USA), 100 U/mL of Penicillin and 100 µg/mL of Streptomycin (P/S) (Gibco^®^, Life Technologies, Carlsbad, CA, USA) and incubated at 37 °C in a 5% CO_2_ atmosphere. Peripheral blood mononuclear cells (PBMCs) were isolated by Ficoll-hypaque (Lonza Group, Basel, Switzerland) from healthy donors, following informed consent and Institutional Review Board (University of Catanzaro, Catanzaro, Italy) approval (institutional approval: n.266/2021). PBMCs were cultured in RPMI-1640 medium (Gibco^®^, Life Technologies) supplemented with 20% fetal bovine serum (Lonza Group Ltd., Basel, Switzerland) and 1% penicillin/streptomycin (Gibco^®^, Life Technologies), as reported [70]. The 293T cell line was purchased from DSMZ and cultured in DMEM (Gibco^®^, Life Technologies) supplemented with 10% fetal bovine serum (Lonza Group Ltd.) and 1% penicillin/streptomycin (Gibco^®^, Life Technologies).

The selective HDAC6 inhibitor **ACY-1215** (HY-16026) was purchased from MedChemExpress (MCE, Monmouth Junction, NJ, USA).

### 4.6. Cell Viability and Proliferation Assay

Cell viability was assessed using the CellTiter-Glo (CTG) assay kit (Promega, Madison, WI, USA), as reported [32]. Briefly, cells were seeded in 24-well plates and treated with **Compound 10** at various concentrations. Luminescence was measured using the GloMax-Multi Detection System (Promega, Madison, WI, USA).

### 4.7. HDAC6 Inhibition In Vitro Assay

HDAC6 enzymatic activity was assessed using a fluorogenic HDAC6 assay kit (BPS Bioscience, cat#50076, San Diego, CA, USA), which includes a fluorometric substrate containing an acetylated lysine side chain. Upon deacetylation by HDAC6, the substrate becomes reactive to the Lysine Developer, which generates a fluorophore detectable using a fluorescence reader. Compounds AKOS030273637 (**10**), AKOS030461429 (**9**), AKOS000531201 (**8**), **ACY-1215** and **Trichostatin A** were diluted in HDAC assay buffer and mixed with HDAC substrate (200 µM), BSA (1 mg/mL), HDAC assay buffer and HDAC6 human recombinant enzyme (14 ng/µL). The reaction mixtures were incubated at 37 °C for 30 min, and then the HDAC developer was added, and the mixtures were incubated at room temperature for 15 min. Fluorescence was measured using the GloMax-Multi Detection System (Promega, Madison, WI, USA). The dose–response curve and IC_50_ values were determined using GraphPad Prism based on increasing concentrations (0.1, 0.25, 0.5, 1.0, 2.5, 5.0, 10.0, 25.0 and 50.0 mM) of **Compound 10**.

### 4.8. Western Blotting (WB) Assays

Total protein extracts were prepared using NP-40 Cell Lysis Buffer supplemented with Halt™ Protease Inhibitor Cocktail (Thermo Fisher Scientific, Waltham, MA, USA), as reported [70].

Protein samples (30 µg per lane) were resolved on 10% SDS-PAGE gels and subsequently transferred onto nitrocellulose membranes using the Trans-Blot Turbo Transfer System (Bio-Rad Laboratories, Hercules, CA, USA). Membranes were then blocked and incubated with the following primary antibodies: acetylated-α Tubulin (#sc-23950; Santa Cruz Biotechnology, Dallas, TX, USA), acetylated-histone H4 (#sc-34263; Santa Cruz Biotechnology) or histone H4 (#AB5823; abcam). GAPDH (MA5-15738; Thermo Fisher Scientific, Waltham, MA, USA) was used as loading control. Protein bands were visualized using the Clarity Western ECL Substrate (Bio-Rad Laboratories, Hercules, CA, USA), and chemiluminescence signals were quantified using the UVITEC Imaging System (Cleaver Scientific, Warwickshire, UK).

## 5. Conclusions

Through an integrated computational and experimental approach, **Compound 10** was identified as a promising HDAC6 inhibitor with favorable drug-like properties and stable binding behavior. It showed strong inhibitory in vitro activity and demonstrated anti-tumor effects in MM models, particularly in proteasome inhibitor-sensitive cells. The compound increased α-tubulin acetylation, supporting its on-target action. These findings suggest that **Compound 10** represents a promising lead for further development in MM therapy. However, additional studies are required to assess its isoform selectivity profile, in vivo efficacy, and potential predictive biomarkers of response. Moreover, a comprehensive evaluation of its pharmacokinetic, pharmacodynamic, and toxicity profiles will be essential to support its progression toward preclinical development. Future work will focus on these aspects to better define its therapeutic potential.

## Figures and Tables

**Table 1:** **Representative “*Training Set*” compounds used for shared pharmacophore generation.** These six structurally different HDAC6 inhibitors were selected as cluster centroids based on Tanimoto similarity and served as the basis for constructing the ligand-based pharmacophore model.

Compound	Structure
**Compound 1**	** 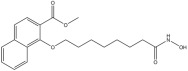 **
**Compound 2**	** 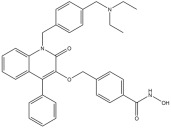 **
**Compound 3**	** 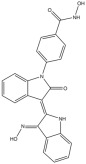 **
**Compound 4**	** 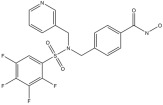 **
**Compound 5**	** 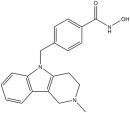 **
**Compound 6**	** 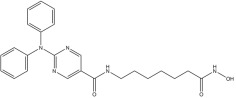 **

**Table 2:** Glide score (G-Score) and AKOS code of the selected compounds and the reference inhibitor **Trichostatin A** in complex with HDAC6. The G-score values are reported in kcal/mol.

Compound	AKOS Code	G-Score (kcal/mol)
**Compound 7**	AKOS030496586	−6.49
**Compound 8**	AKOS000531201	−7.18
**Compound 9**	AKOS030461429	−9.87
**Compound 10**	AKOS030273637	−8.04
**Trichostatin A**		−5.52

**Table 3:** HDAC6 inhibitory activity of **Compound 8**, **Compound 9**, and **Compound 10**. IC_50_ values (mean ± SD) were calculated using GraphPad Prism software v. 10.2.0 from three independent experiments. **ACY-1215** and **Trichostatin A** were included as positive controls.

Compound	HDAC6 IC_50_ (μM)
**Compound 10**	1.69 ± 0.42
**Compound 8**	>10
**Compound 9**	>25
**ACY-1215**	0.44 ± 0.41
**Trichostatin A**	0.43 ± 0.70

## Data Availability

Data is contained within the article or Supplementary Materials.

## Supplementary Materials

The following supporting information can be downloaded at: https://www.mdpi.com/article/10.3390/ph18091303/s1, Table S1. List of 31 active compounds used as the training set for ligand-based pharmacophore modeling; Table S2. Pharmacophores; **Figure S1.** ***Pharmacophore models mapped onto the training set****compounds;*** Figure S2. Clustering analysis; Figure S3. RMSD Analysis of HDAC6–Ligand Complexes; Figure S4. Protein–ligand interactions between **Trichostatin A** and HDAC6 residues; **Table S3. List of active compounds used for pharmacophore validation;** Table S4. List of decoy compounds generated from the DUD-E database; **Table S5.** List of inactive compounds retrieved from ChEMBL; **Figure S5. Dose–response curve analysis of HDAC6 activity in the presence of increasing concentrations of Compound 10. Figure S6**. Heatmap showing cell viability assessed by the CellTiter-Glo assay in MM cell lines treated with **ACY-1215** or vehicle control (DMSO) for 72 h; Figure S7. Cell viability assessed by the CellTiter-Glo assay in 293T (A) or healthy PBMCs (B), 72 h after treatment with **Compound 10**; Figure S8. Western blot analysis of acetylated α-tubulin in AMO and AMO-BZB cells, 48 h after treatment with **ACY-1215**; Figure S9. Western Blot Analysis of Histone H4 and α-Tubulin Acetylation in AMO Cells Treated with **Compound 10** and ACY1215; **Figure S10. Three-dimensional visualization of redocking analysis.**

## Abbreviations

The following abbreviations are used in this manuscript:

AUC	Area under the curve
BSA	Bovine Serum Albumin
BZB	Bortezomib
CD1	Catalytic domain 1
CD2	Catalytic domain 2
DMSO	Dimethyl Sulfoxide
DNA	Deoxyribonucleic Acid
DUD-E	Directory of Useful Decoys, Enhanced
FBS	Fetal Bovine Serum
FDA	Food and Drug Administration
GAPDH	Glyceraldehyde 3-Phosphate Dehydrogenase
HBA	Hydrogen bond acceptor
HBD	Hydrogen bond donor
HDAC	Histone Deacetylase
HDACis	Histone Deacetylase Inhibitors
HY	Ionizable group
IC_50_	Half maximal inhibitory concentration
IUPAC	International Union of Pure and Applied Chemistry
K	Kelvin
LBPS	Ligand-Based Pharmacophore Screening
LBVS	Ligand-Based Virtual Screening
MCE	MedChemExpress
MDs	Molecular Dynamic simulation
MM	Multiple Myeloma
MM-GBSA	Molecular Mechanics Generalized Born Surface Area
MTK_NPT	Martyna-Tobias-Klein for NPT ensemble
μM	Micromolar
NF-kB	Nuclear Factor kappa-light-chain-enhancer of activated B cells
NI	Hydrophobic feature
ns	Nanosecond
OPLS	Optimized Potentials for Liquid Simulations
PDB	Protein Data Bank
PIs	Proteasome Inhibitors
PME	Particle Mesh Ewald
r-RESPA	Reversible Reference System Propagation Algorithm
RMSD	Root Mean Square Deviation
ROC	Receiver Operating Characteristic
SBVS	Structure-Based Virtual Screening
SID	Simulation Interaction Diagram
SP	Standard Precision
TIP4P	Transferable Intermolecular Potential with 4 Points
TSA	**Trichostatin A**
VS	Virtual Screening
ZBG	Zinc-binding group
ZnF UBP	Zinc finger ubiquitin-binding domain
