# Supplementary material for: Discovery of a Promising Hydroxyamino-Piperidine HDAC6 Inhibitor via Integrated Virtual Screening and Experimental Validation in Multiple Myeloma
Source: Pharmaceuticals (Basel). 2025 Aug 29;18(9):1303. doi: 10.3390/ph18091303 (PMC12472302; doi:10.3390/ph18091303)
Supplement: Supplementary file 1 [file pharmaceuticals-18-01303-s001.zip › pharmaceuticals-3759629-supplementary.pdf]

Supplementary Material for

Discovery of a Promising Hydroxyamino-Piperidine HDAC6 Inhibitor via Integrated Virtual Screening and Experimental Validation in Multiple Myeloma

Federica Chiera [1,†], Antonio Curcio [1,†], Roberta Rocca [1,2,*], Ilenia Valentino [3], Massimo Gentile [4,5], Stefano Alcaro [1,2], Nicola Amodio [3,*] and Anna Artese [1]

[1] Dipartimento di Scienze della Salute, Università Magna Græcia, 88100 Catanzaro, Italy; f.chiera@unicz.it (F.C.); antonio.curcio@unicz.it (A.C.); alcaro@unicz.it (S.A.); artese@unicz.it (A.A.)
[2] Associazione CRISEA-Centro di Ricerca e Servizi Avanzati per l'Innovazione Rurale, Località Condoleo di Belcastro, 88100 Catanzaro, Italy
[3] Dipartimento di Medicina Sperimentale e Clinica, Università Magna Græcia, 88100 Catanzaro, Italy; ilenia.valentino30@gmail.com
[4] Hematology Unit, Azienda Ospedaliera Annunziata, 87100 Cosenza, Italy; massimo.gentile@unical.it
[5] Department of Pharmacy, Health and Nutritional Science, University of Calabria, 87036 Rende, Italy
* Correspondence: rocca@unicz.it (R.R.); amodio@unicz.it (N.A.)
† These authors equally contributed to this study.

Table S1. List of 31 active compounds used as the training set for ligand-based pharmacophore modeling. Compounds were selected based on their potent antitumor activity (HDAC6 IC_{50} values < 100 nM) and documented selectivity toward HDAC6 across various cancer models. For each compound, the IC_{50} values and corresponding literature references have been included to support the selection criteria.

Table S2. Pharmacophores. Ten pharmacophore models with their corresponding features, Fit Scores, and Atom Overlap values. Feature abbreviations: HBD – Hydrogen Bond Donor; HBA – Hydrogen Bond Acceptor; NI – Negatively Ionizable Area; H – Hydrophobic Interaction.

Figure S1. Pharmacophore models mapped onto the training set compounds. Depiction of the shared pharmacophoric features identified across the structurally diverse training set used for model generation, comprising: 1) Compound 1, 2) Compound 2, 3) Compound 3, 4) Compound 4, 5) Compound 5 and 6) Compound 6. The consensus pharmacophore includes two hydrogen bond acceptors (HBA), one hydrogen bond donor (HBD), one hydrophobic feature (HY), and one negatively ionizable group (NI). The alignment highlights the conserved spatial arrangement of these features, which are critical for effective HDAC6 binding.

Figure S2. Clustering analysis. Clustering of the 19 selected hits based on fingerprint similarity using MACCS (Molecular ACCess System) keys and the Tanimoto coefficient. Compounds were grouped by fingerprint similarity, and a representative centroid was chosen for each cluster as the compound sharing the greatest number of features with others in the group.

Figure S3. RMSD Analysis of HDAC6–Ligand Complexes. Root Mean Square Deviation (RMSD) plots of the heavy atoms of HDAC6 in complex with selected candidate compounds and Trichostatin A. RMSD values, expressed in Ångström (Å), were monitored throughout the molecular dynamics simulations to evaluate the structural stability of each protein–ligand complex over time.

Figure S4. **Protein–ligand interactions between Trichostatin A and HDAC6 residues.** Only interactions occurring for more than 30% of the 200 ns simulation time are displayed.

Table S3. List of active compounds used for pharmacophore validation.

Table S4: List of decoy compounds generated from the DUD-E database.

Table S5. List of inactive compounds retrieved from ChEMBL.

Figure S5. Dose-response curve analysis of HDAC6 activity in the presence of increasing concentrations of Compound 10. Enzymatic activity is expressed as a percentage of residual activity relative to control (DMSO).

Figure S6. Heatmap showing cell viability assessed by the CellTiter-Glo assay in MM cell lines treated with ACY-1215 or vehicle control (DMSO) for 72 hours. Viability is expressed as a percentage relative to vehicle-treated cells. The half-maximal inhibitory concentrations (IC_{50}) of ACY-1215 for AMO, AMO-BZB, H929, and H929-BZB cell lines are reported in the accompanying table. IC_{50} values (mean ± SD) were calculated using GraphPad Prism software based on three independent experiments.

Figure S7. Cell viability assessed by the CellTiter-Glo assay in 293T (A) or healthy PBMCs (B), 72 hours after treatment with Compound 10. Viability is expressed as a percentage relative to vehicle-treated cells.

Figure S8. Western blot analysis of acetylated α-tubulin in AMO and AMO-BZB cells, 48 hours after treatment with ACY-1215. GAPDH was used as a loading control.

Figure S9. Western Blot Analysis of Histone H4 and α-Tubulin Acetylation in AMO Cells Treated with Compound 10 and ACY1215. A) Western blot analysis of acetylated- H4 and histone H4 in AMO cells treated for 72 hours with Compound 10. **B**) Normalized expression of acetylated-H4 and acetylated- α-tubulin in AMO cells, 72 hours after treatment with Compounds 10. **C**) Western blot analysis of acetylated- H4 and histone H4 in AMO cells treated for 48 hours with ACY1215. **D**) Normalized expression of acetylated-H4 and acetylated- α-tubulin in AMO cells, 48 hours after treatment with Compounds 10. GAPDH was used a loading control. *p<0.05 compared to corresponding vehicle.

Figure S10. Three-dimensional visualization of redocking analysis. Superimposition of the docked ligand pose with the crystallographic conformation of the reference ligand within the HDAC6 active site. For chain A, the docked ligand is shown in light blue, for chain B, in pink.

SMILES	Cancer Model	HDAC6 IC_{50} (nM)	PMID / DOI
ONC(=O)c1ccc(cc1)CNc2cccc(c23)cccn3	Multiple myeloma	0.291	35745586
ONC(=O)CCCCO\N=C\c(c1)cnc(c12)cccc2	Ovarian carcinoma	10	33100043
ONC(=O)c1ccc(cc1)CN(S(=O)(=O)c(c2F)cc(F)c(F)c2F)Cc3cccnc3	Leukemia	2.1	33576627
ONC(=O)c1ccc(cc1)CN2CC(=O)N(Cc3ccccc3)[C@@H](C2=O)Cc4ccccc4	hematological tumor	9.83	33576627
ONC(=O)c1ccc(cc1)CN2CC(=O)N[C@H](C2=O)CC.C=CCC	Colon cancer	0.73	33576627
ONC(=O)c1ccc(cc1)Cn(c(c23)cccc3)c(=O)n(c2=O)CCc4ccccc4	Lung cancer	4	37108796
ONC(=O)/C=C/c(cc1)cc(c12)ncn(c2=O)CC3CCCCC3	Colon cancer	12	33644598
ONC(=O)c1ccc(cc1)Cc(cc2)cc(c2c34)nc(n4nc(n3)N)Cc(c5)ccc(c56)OCO6	Liver cancer	0.5	10.1039/D2RA01753A
O1COc(c12)ccc(c2)CC(=O)Nc(cc3)c(-c4nc(N)n[nH]4)cc3Cc(cc5)ccc5C(=O)NO	Liver cancer	0.1	10.1039/D2RA01753A
ONC(=O)c1ccc(cc1)CN(OCCC)C(=O)Nc(c2)cc(C)cc2C	Hematological malignancy	14	33629513
ONC(=O)c1ccc(cc1)CN(CCCCO)C(=O)Nc(cc2)ccc2CN	Melanoma	0.4	32815366

**ONC(=O)/C=C/c1ccc(cc1)NC(=O)N(CC2)CCC2NC(=O)c3ccc(cc3)OC**	Breast cancer	12.3	30867374
**ONC(=O)CCCCCNC(=O)c1cnc(s1)-c2c(OC)cc(cc2)OC**	Melanoma	31	35043615
**ONC(=O)CCCCCNC(=O)c1cn(nn1)C2CCN(CC2)C(=O)c3ccc(cc3)N(C)C**	Breast cancer	11.5	31019142
**ONC(=O)CCCCCNC(=O)c1cn(nn1)C2CCN(CC2)S(=O)(=O)c(c3)ccc(c34)cccc4**	Breast cancer	8.6	31019142
**ONC(=O)CCCCCNC(=O)c1cn(nn1)Cc2ccc(cc2)NS(=O)(=O)c3ccc(Br)cc3**	Melanoma	8.4	31155552
**ONC(=O)c1ccc(cc1)NCc2[nH]c(-c(s3)ccc3C)n[n+]2-c4ccccc4**	Gastric cancer	30.6	33831778
**ONC(=O)c1ccc(cc1)CN(C(=O)c2c(C(F)(F)F)cccc2)Cc3nnnn3Cc4ccccc4**	Leukemia	30	32803970
**ONC(=O)c1ccc(cc1)CN2C(=O)N(Cc3cccnc3)[C@@H](C2=O)Cc4ccccc4**	Leukemia	2.1	33992929
**ONC(=O)c1ccc(cc1)N(c(c23)cccc3)C(=O)C\2=C(\C\4=N\O)Nc(c45)cccc5**	Colon cancer	7	32916298
**ONC(=O)c1ccc(cc1)CN2c(nccc3)c3Sc(c24)cccc4**	Breast adenocarcinoma	5	30645113
**ONC(=O)c1ccc(cc1)COc(c2-c3ccccc3)c(=O)n(c(c24)cccc4)Cc(cc5)ccc5CN(CC)CC**	Colon cancer	6.9	33199154
**ONC(=O)c1ccc(cc1)COC(=O)N(CC2)CCC23c4c(cccc4)N(C3)Cc5ccccc5**	Multiple myeloma	48.5	33214839
**ONC(=O)CCCCCCC[C@@H](C1=O)c(cccc2)c2CN([C@@H]13)C(=O)CC3**	Breast cancer	21.42	34153811
**c1ccccc1C[C@@H](C(=O)NO)CC(=O)Cc2cccc(c23)cccc3**	Breast cancer	88	31223439
**ONC(=O)CCCCCCCOc1c(C(=O)OC)ccc(c12)cccc2**	Mesothelioma	95	31223439
**ONC(=O)c1ccc(cc1)CNc2ncnc(c23)[nH]cc3**	Multiple myeloma	10	34474303
**C1=CC=C(C=C1)N(C2=CC=CC=C2)C3=NC=C(C=N3)C(=O)NCCCCCCC(=O)NO**	Myeloma	10	30987296
**CN1CCC2=C(C1)C3=CC=CC=C3N2CC4=CC=C(C=C4)C(=O)NO**	Myeloma	15	36127263
**CCCCN(CC1=CC=C(C=C1)C(=O)NO)C(=O)NC2=CC=CC=C2**	Myeloma	5.02	23009203

Table S1. List of 31 active compounds used as the training set for ligand-based pharmacophore modeling. Compounds were selected based on their potent antitumor activity (HDAC6 IC_{50} values < 100 nM) and documented selectivity toward HDAC6 across various cancer models. For each compound, the IC_{50} values, the related cancer model and the corresponding literature references (PMID or DOI) have been included to support the selection criteria.

Table S2. Pharmacophores. Ten pharmacophore models with their corresponding features, Fit Scores, and Atom Overlap values. Feature abbreviations: HBD – Hydrogen Bond Donor; HBA – Hydrogen Bond Acceptor; NI – Negatively Ionizable Area; HY – Hydrophobic Interaction.

Model	Features	Pharmacophore fit and atom overlap
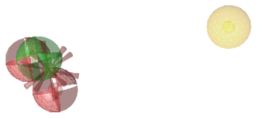	NI, HBD, 2HBA, HY	0.8347
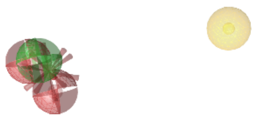	NI, HBD, 2HBA, HY	0.8341

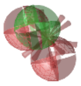	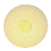	NI, HBD, 2HBA, HY	0.8327
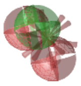	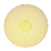	NI, HBD, 2HBA, HY	0.8317
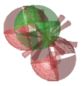	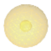	NI, HBD, 2HBA, HY	0.8315
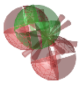	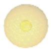	NI, HBD, 2HBA, HY	0.8305
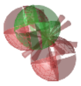	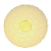	NI, HBD, 2HBA, H	0.8304
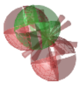	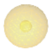	NI, HBD, 2HBA, HY	0.8291

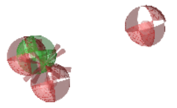	NI, HBD, 3HBA	0.8189
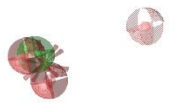	NI, HBD, 3HBA	0.8121

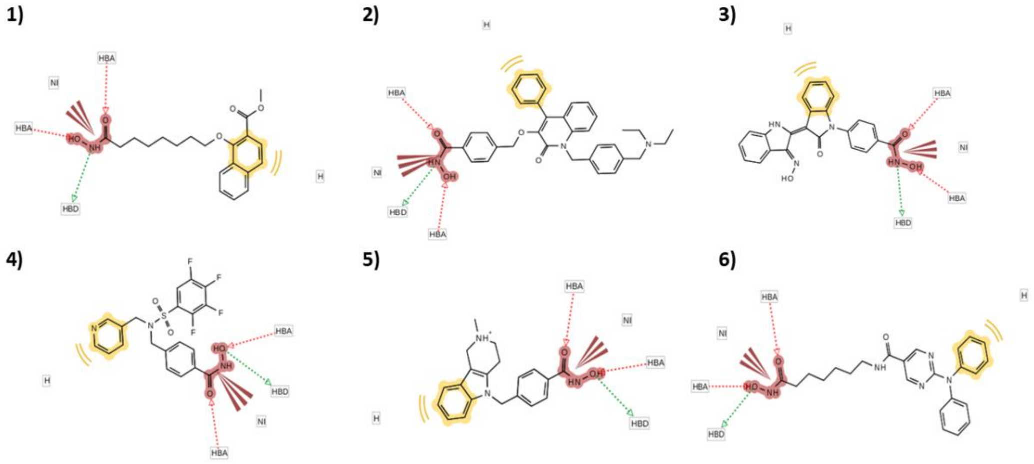

Figure S1. Pharmacophore models mapped onto the training set compounds. Depiction of the shared pharmacophoric features identified across the structurally diverse training set used for model generation, comprising: **1) Compound 1**, **2) Compound 2**, **3) Compound 3**, **4) Compound 4**, **5) Compound 5** and **6) Compound 6**. The consensus pharmacophore includes two hydrogen bond acceptors (HBA), one hydrogen bond donor (HBD), one hydrophobic feature (HY), and one negatively ionizable group (NI). The alignment highlights the conserved spatial arrangement of these features, which are critical for effective HDAC6 binding.

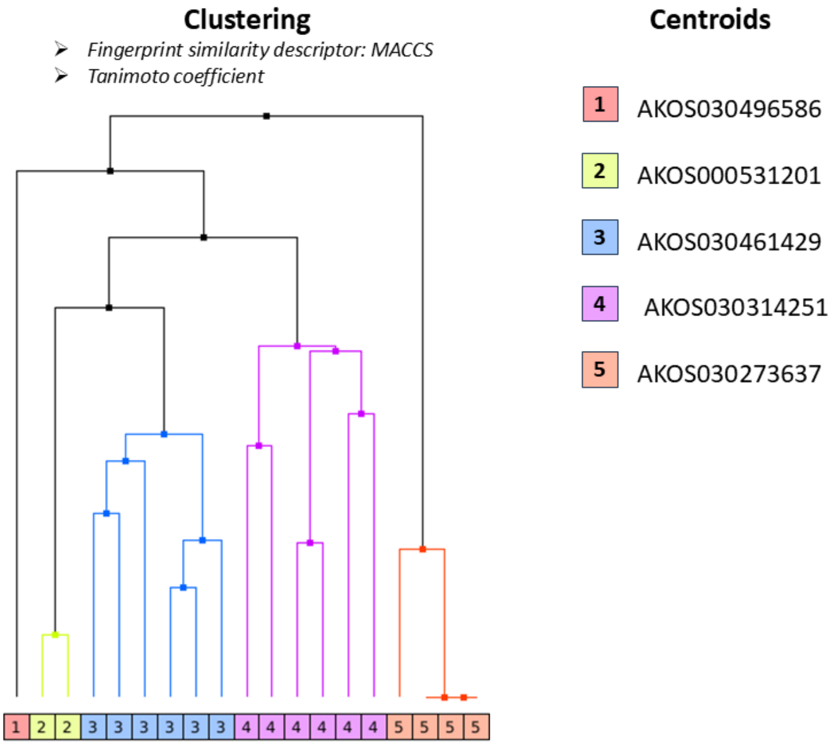

Figure S2. Clustering analysis. Clustering of the 19 selected hits based on fingerprint similarity using MACCS (Molecular ACCess System) keys and the Tanimoto coefficient. Compounds were grouped by fingerprint similarity, and a representative centroid was chosen for each cluster as the compound sharing the greatest number of features with others in the group.

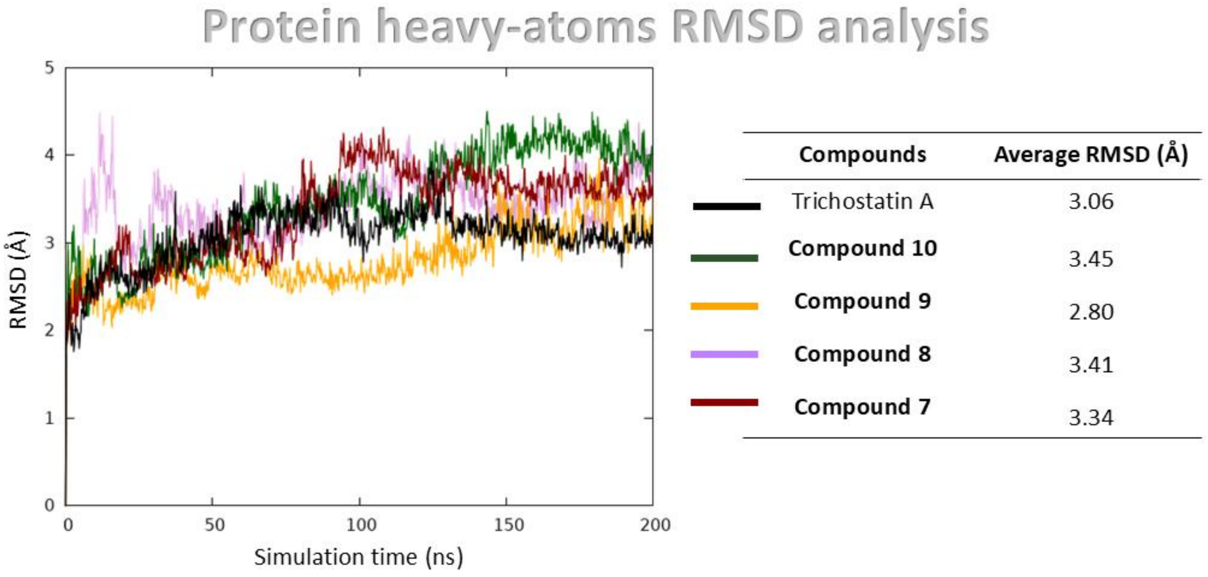

Figure S3. RMSD Analysis of HDAC6–Ligand Complexes. Root Mean Square Deviation (RMSD) plots of the heavy atoms of HDAC6 in complex with selected candidate compounds and Trichostatin A. RMSD values, expressed in Ångström (Å), were monitored throughout the molecular dynamics simulations to evaluate the structural stability of each protein–ligand complex over time. Moreover, the average RMSD values for each complex are summarized in the table.

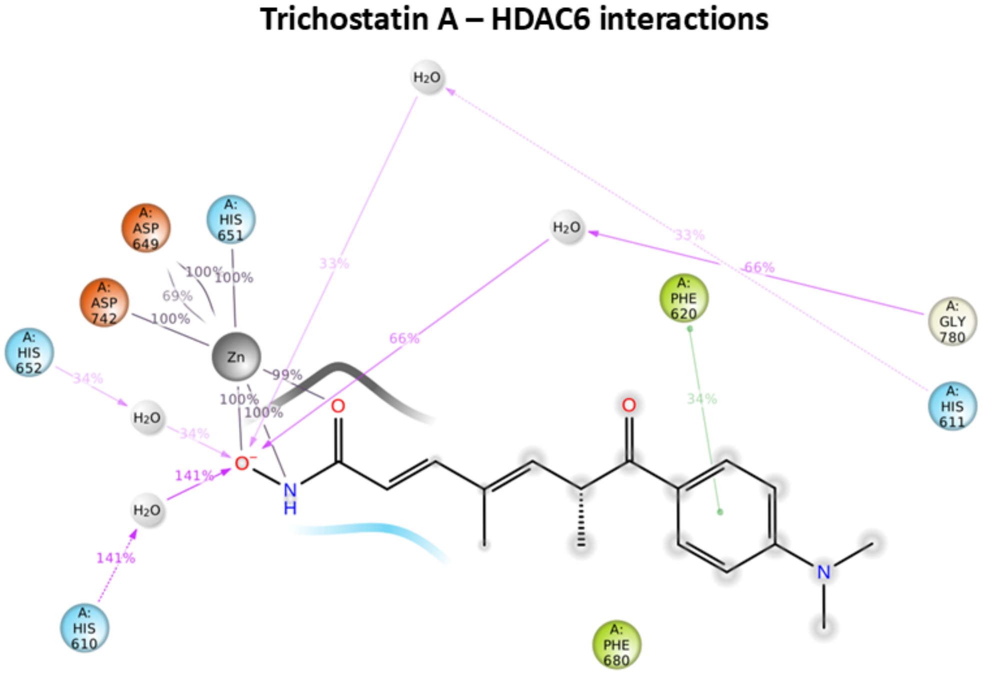

Figure S4. Protein–ligand interactions between Trichostatin A and HDAC6 residues. Only interactions occurring for more than 30% of the 200 ns simulation time are displayed.

Table S3. List of active compounds used for pharmacophore validation.

**Active compound SMILES (Simplified Molecular Input Line Entry System)**
C1=CC=C(C=C1)N(C2=CC=CC=C2Cl)C3=NC=C(C=N3)C(=O)NCCCCCCC(=O)NO
C1=CC(=CC=C1CN(C2=NC=CN=C2)C3=NC=CN=C3)C(=O)NO
C1=CC=C(C=C1)N(CCO)C(=O)CC2=CC=C(C=C2)C(=O)NO
C1=CC=C(C=C1)CCN2C(=O)C3=CC=CC=C3N(C2=O)CC4=CC=C(C=C4)C(=O)NO
C1CC1(C2=CC=CC=C2)NC3=NC=C(C=N3)C(=O)NO
C1=CC=C(C=C1)N(C2=CC=CC=C2)C3=CC=C(C=C3)/C=N/NC(=O)CCCCCCC(=O)NO
C1=CC(=C(C=C1C(=O)NCCC2=CC(=NO2)C(=O)NO)Cl)Cl
C1=CC(=CC=C1CNC(=O)C2=C(C=C(N2)C3=CC=C(C=C3)O)C4=COC=C4)C(=O)NO
CC1=NC2=CC=CC=C2C(=N1)N(C)C3=CC(=C(C=C3)OC)OCCCC(=O)NO
CN1C=CC=C1C(=O)N2CCC3=C(C2)C=CC(=C3)C(=O)NO
C1=CC(=CC=C1CNC(=O)C2=C(C(=C(C(=C2F)F)F)F)F)C(=O)NO
COC(CC1)CCC1C(=O)C(C2)CCC(C23)N(CC3)CC(CC4)CCC4C(=O)NO

Table S4: List of decoy compounds generated from the DUD-E database.

**Decoy compound SMILES (Simplified Molecular Input Line Entry System)**
C[C@H](/C=C(\C)/C=C/C(=O)NO)C(=O)c1ccc(cc1)N(C)C
COc1ccc(cc1)NC[C@@H](c2ccc3c(c2)OCO3)O
CCc1c(nn(c1N)[C@@H]2CCS(=O)(=O)C2)CCC(C)C

Cc1c(cnn1C)[C@@H](C)[NH2+][C@H](C)c2ccc(cc2[O-])OC
CCn1cnc2c1ccc(c2)NCc3cccc(c3O)OC
CC(C)[C@H](C(=O)OC)NC(=O)[C@H]1C[C@@H]2CCCC[C@@H]2N1
CC(C)Oc1cccc(c1)[C@@H](c2nc(on2)C(F)(F)F)N
CC[C@](C)(c1nc(on1)c2ccccc2OC(F)F)N
Cc1nnc(n1N)SCCOc2ccc3ccccc3c2
CCNC(=O)Nc1cc(ccc1OC[C@@H]2CCCO2)Cl
COC(=O)c1cc(c(cc1N)F)OC[C@@H]2CCCCO2
CCc1cccc(c1)[N-]S(=O)(=O)c2ccc[nH+]c2NC
CCN1C(=[NH2+])[C@@H](N=C1C2CC2)c3cccc(c3OC)OC
C[C@H]1CCc2cccc(c2N1)NS(=O)(=O)CCOC
CCC1(CCN(CC1)C(=O)NC(C)(C)CCC(=O)O)C
CCC1=NO[C@H](C1)CNCc2[nH]c3c(c(ccc3n2)C)C
Cc1cc(n(n1)C[C@@H](C)NCc2c3c([nH]n2)CCCC3)C
CCCCN(C)S(=O)(=O)N1CCCc2c1ccc(c2)N
CC[C@@H]1C[C@H](CCO1)Nc2ccccc2NS(=O)(=O)C
CCN(CC(=O)NC1(CCCCCC1)C#N)[C@@H]2CCNC2
Cc1cc2nc(cc(n2n1)NCC3(CCC3)CO)C(C)C
CC(C)CNC(=O)NCCc1cc(cc2c1OCOC2)F
Cc1cc(ccc1NCc2c(nc(s2)N(C)C)OC)O
CCC1CCC(CC1)CNC(=O)N2CC(C2)CC(=O)O
CCC[NH2+][C@H]1CCCC[C@H]1Sc2c3c([n-]cn3)ncn2
C[C@H](CNc1cc(ccc1[N+](=O)[O-])Br)CO
CC(C)(CC(=O)O)NC(=O)N(C)C[C@H]1C[C@H]2CC[C@@H]1C2
CC[C@H]1C(=O)N(CC(=O)N1)c2ccc(cc2)[NH+](CC)CC
c1cc(c(cc1[N+](=O)[O-])Cl)CNCC2(CCCC2)O
CCc1c(c(on1)CC)CNC(=O)N[C@@H]2CCC[C@H](C2)C
C[C@@H]1C[C@H](CN(C1)C(=O)C[NH2+]C(C)(C)C(C)(C)C(=O)[O-])C
CCCN1C(=N[C@@H](C1=[NH2+])c2ccc(c(c2)C#N)OC)CC
C[C@H](c1ncc(o1)C(C)(C)C)O/N=C(\c2ccsc2)/N
c1cc(c(cc1Cl)[N+](=O)[O-])CNC[C@H]2CCC[C@@H]2O
CCCn1cncc1[C@@H]2C(=[NH2+])N(C(=N2)C(C)(C)C)CC#C
CC(C)CN1C(=[NH2+])[C@H](N=C1C2CC2)c3cncn3C(C)C
C[C@@H](CC(C)C)NC(=O)NC[C@@H]1CN2CCCC[C@@H]2CO1
Cc1cc(cc(c1)F)NCc2ccc(o2)S(=O)(=O)NC
CC(C)c1c(c(n(n1)C)N(C)Cc2ccccc2OC)N
CCCc1c(nn(c1N)[C@H]2CCS(=O)(=O)C2)[C@@H](C)CC
Cc1cc(=O)[nH]c(n1)SCC[C@@H]2CCC[C@@]2(C#N)NC
CCCN[C@@]1(CCC[C@@H](C1)Sc2[nH]c(=O)ccn2)C#N
CCCN[C@@]1(CCC[C@H](C1)Sc2[nH]ccc(=O)n2)C#N
CCC[C@]1(CCC[NH2+]C1)c2[n-]c3ccc(nc3n2)OCC
[H]/N=C/1\[C@@](N(C(=O)N1)CC(C)C)(C)Cc2ccccc2OC
CCCOc1c(c(ncn1)Sc2nc(cs2)C)N
CCCN[C@H](c1ccc2c(c1)oc(=O)[nH]2)[C@]3(CCCO3)C
C[C@H](CNC(=O)NCC1CCS(=O)(=O)CC1)C(C)C
C[C@]1(CCOC1)NC(=O)Nc2ccc(cc2)OCC3CC3
Cc1c(c(n(n1)C(C)C)N(C)Cc2ccc(cc2)OC)N
CC(C)(C)NC(=O)Nc1cccc(c1)OC[C@@H]2CCCO2
c1ccc(cc1)N(c2ccccc2)c3ncc(cn3)C(=O)NCCCCCCC(=O)NO
COc1ccc(cc1)OC[C@H](CNC(=O)Nc2ccc(cc2)OC(F)(F)F)O
COc1ccccc1C(=O)N[C@](C(=O)OC)(C(F)(F)F)NNc2ccc(cc2)F
CCOC(=O)N[C@H](C(Cl)(Cl)Cl)NC(=S)Nc1cccc(c1)[N+](=O)[O-]
CCCCCCn1c(nnc1S[C@@H](C)C(=O)NC(=O)N)c2ccc(cc2)Cl

CCCc1nnc(n1N)SCC(=O)Nc2c(c3c(s2)C[C@H](CC3)C)C(=O)OCC
CCc1c(sc(c1C(=O)OCC)NC(=O)COC(=O)c2ccc(c(c2)N)Cl)C
CCOC(=O)c1c(c(c(s1)N)C#N)CSc2[nH]c(nn2)CCC3CCCC3
COc1ccccc1C(=O)N[C@](C(=O)OC)(C(F)(F)F)NNc2cccc(c2)Cl
Cc1ccccc1OCC(=O)NC(=S)NNC(=O)COc2ccc(cc2C)Cl
CC(C)CCN(CCOc1ccccc1F)c2c(n(c(=O)[nH]c2=O)Cc3ccccc3)N
CC[C@@H](C)c1ccccc1OCC(=O)NNC(=S)NC(=O)c2ccc(o2)Br
CCCOc1ccccc1C(=O)NC(=S)NNC(=O)COc2ccccc2Cl
CCCOc1cccc(c1)C(=O)NC(=S)NNC(=O)COc2ccc(cc2Cl)Cl
CCCOc1ccccc1C(=O)NC(=S)NNC(=O)Cn2ccc3c2cccc3
C[C@H](C(=O)Nc1c(ccs1)C(=O)N)OC(=O)COc2ccc(cc2)C(C)(C)C
Cc1cc(c(cc1Cl)OC)NC(=O)[C@H](C)OC(=O)c2ccccc2NCCO
Cc1ccc(c(c1)OCc2nnc(n2N)S[C@@H](C)C(=O)Nc3ccc(cc3)OC)C(C)C
CC/C(=N/Nc1nnc(n1N)SCC(=O)c2cccc(c2)OC)/c3ccccc3
CCOc1ccc(cc1)/C=C/C(=O)OCC(=O)Nc2c(c3c(s2)CCCC3)C(=O)N
Cc1cccc(c1C)NC(=S)/N=C(/NCCN2CCOCC2)\Nc3nc(cc(n3)C)C
Cc1ccc(c(c1)OCc2nnc(n2N)S[C@@H](C)C(=O)NCc3cccs3)C(C)C
CCOc1ccc(cc1)NS(=O)(=O)c2ccc(c(c2)C(=O)NNC(=O)CC(C)C)Cl
CCCCN(c1c(n(c(=O)[nH]c1=O)CC(C)C)N)C(=O)[C@@H](C)Oc2ccc(cc2C)Cl
C[C@H](c1ccc(cc1)F)NC(=O)CSc2nnc(n2N)COc3ccc(cc3)C(C)(C)C
C[C@H](C(=O)NCCNc1ccc(cc1[N+](=O)[O-])C(F)(F)F)NC(=O)C(C)(C)C
C/C=C\[C@H](C(=O)NC(C)(C)CC(C)(C)C)N(Cc1ccc(o1)C)C(=O)c2cccc(c2O)O
CCCCN(c1c(n(c(=O)[nH]c1=O)CC(C)C)N)C(=O)COc2cc(ccc2Cl)Cl
CCOc1cc(c(cc1NC(=O)CC(C)C)OCC)NC(=S)NCc2ccco2
CC1=CC(=C)NC(=O)[C@H]1CNC(=O)[C@@H](CC(C)C)NS(=O)(=O)c2ccc(cc2)OC
CCCCSc1nc2n(n1)[C@H](C(=C(N2)C)C(=O)N)c3ccc(c(c3)OCC)OC
CCCn1c(c(c(=O)[nH]c1=O)C(=O)CN(CCC)[C@@H](C)c2ccccc2Br)N
CC(C)CC(=O)Nc1ccc(cc1)NC(=O)NC[C@H](c2ccccc2OC)N3CCCC3
CCN(CC)c1ccc(c(c1)O)/C=N/Nc2[nH]nc(n2)SCc3ccc(cc3)F
CCN(CC)c1ccc(cc1)NC(=O)CSc2nc([nH]n2)NN=C3CCCCC3
CCCC(=O)Nc1cccc(c1)NC(=S)NC(=O)c2ccc(cc2OC)OC
c1cc(ccc1NC(=O)CNCCCNc2ccc(cc2[N+](=O)[O-])C(F)(F)F)Cl
Cc1cc(nc(n1)N/C(=N/C(=S)Nc2ccc(cc2OC)OC)/NC3CCCC3)C
COC(=O)c1ccc(cc1)NC(=O)C(=O)NCC(=O)NCC2(CCCC2)c3cccs3
CCc1ccc(cc1)OC[C@@H]2NN[C@H](N2CC)SCc3cc(=O)c4cccc(c4[nH]3)OC
Cc1c(nc([nH]1)SCC(=O)N[C@@H]2[C@H]([C@H](C(=N2)CCSC)C)C#N)Cc3ccc(cc3)O
Cc1[nH]nc(n1)SCCNCC2=C(NN(C2)c3ccccc3F)c4ccc(cc4)OC
CCOc1ccc(cc1)[C@H]2C(=C(NC(=C2C(=O)OCC=C)C)SCC(=O)N)C#N
C[C@@H](c1ccccc1)NC(=O)C(=O)N/N=C\c2ccc(cc2)OCC(=O)NC3CCCCC3
CCOc1ccc(cc1)[C@H](CCC(=O)N/N=C/c2cc(c(c(c2)OC)O)CC=C)O
CCCCn1c(c(c(=O)[nH]c1=O)N(CCC(C)C)C(=O)/C=C\c2ccc(cc2)F)N
COC(=O)C[C@H](c1ccc(cc1)O)c2c([nH]nc2O)Cc3ccc(cc3)OC(F)(F)F
C[C@H](C(=O)N/N=C\c1ccc(c(c1)OC)OCC(=O)N)Oc2ccc(cc2Cl)Cl
COc1cccc(c1)/C=C(/c2ccccc2)\C(=O)OCC(=O)Nc3c(ccs3)C(=O)N
CCCOc1ccc(cc1)c2cc([nH]n2)C(=O)N/N=C\c3ccc(c(c3)OCC)O
c1cc(cc(c1)Cl)[C@H]2C[C@@H]2C(=O)OCCNc3ccc(cc3[N+](=O)[O-])C(=O)N
C[NH+](C)Cc1ccc(cc1)S(=O)(=O)n2ccc(c2)/C=C/C(=O)NO
CC(=C)Cn1c2c(=O)[nH]c(=O)n(c2[nH+]c1SC[C@H](CCl)O)C
CN(C)c1cc[nH+]c2c1c3c(s2)c(ncn3)N(CCO)CCO
CC[C@H](C)[C@@H](C(=O)N1CCC(CC1)C(=O)N2CCC(CC2)C(=O)OC)[NH3+]
CCCCOC[C@@H]1C[C@@]2(C[C@](OC2=O)(C)c3csc([nH+]3)N)C(=O)O1
C[C@H](COC)NC(=O)C1(CC[NH2+]CC1)Cc2cc(no2)[C@@H]3CCOC3
CC[NH+]1CCN(CC1)C(=O)[C@@H](C)Sc2nnc(n2N)C(F)(F)F

CC(C)C[NH+]1CCO[C@@H](C1)CNC(=O)c2cc3c([nH]c2=O)CCCC3=O
c1cc2cccc3c2c(c1)C(=O)N(C3=O)CC[NH+]4CCN(CC4)CC(=O)N
CC[NH+]1CCN(CC1)[C@H](C)CNC(=O)C(=O)Nc2cccc(c2)C#N
C[C@@H](C(=O)NCc1ccc([nH+]c1)N2CCCC2)NC(=O)c3ccoc3
C[NH+](C)CCCN/C=C/1\C(=O)NC(=O)N(C1=O)c2ccccc2Cl
C[C@@H]1C[C@H](C[NH+](C1)CC(=O)c2c(n(c(=O)n(c2=O)C)CC(C)C)N)C
C=CCNC(=O)NC(=O)C[NH+]1CCC(CC1)C(=O)N2CCCCC2
CCN(CC)[C@@H]1[C@H]([C@@H]([C@H]2CO[C@@H]1O2)[NH2+]Cc3ccc4c(c3)OCO4)O
CC[C@H](C)n1c(ccn1)NC(=O)[C@H](C)[NH+]2CCC(CC2)CNC(=O)C
CCCS(=O)(=O)N1CCC(CC1)[NH2+]C[C@H]2CNc3ccnn3C2
CN(C)c1nc(nc(n1)N)CN2CC[NH+](CC2)Cc3ccsc3
C[C@H](CN1CC[NH+](CC1)C)[NH2+]CCn2c(=O)c3ccccc3c(n2)[O-]
CC[C@@H](C)OC(=O)C[C@H]1C(=O)NCCN1C(=O)[C@@H]2CCC[C@@H]([NH2+]2)C
C[C@H]1CCC[C@@H](C1)NC(=O)C[NH+]2CCN(CC2)CC(=O)NCC#N
CC(C)C[C@@H](c1nnc2n1CC[NH2+]CC2)NC(=O)C3CCOCC3
COc1cc2c(cc(c(=O)[nH]2)C[NH+]3CC=C[C@@H]3CO)c(c1OC)OC
CC1(C(=O)N(C(=O)N1)CC[NH+]2CCN(CC2)C[C@@H](C(C)(C)C)O)C
C[C@@H](CNC(=O)C(=O)Nc1ccccc1OC)CN2CC[NH+](CC2)C
Cc1c(c(nc2c1c(=O)[nH]n2C)C)CC(=O)NCC(C)(C)[NH+](C)C
CCN(CC(=C)C)C(=O)C[NH+]1CCC[C@H](C1)[C@@]2(C(=O)NC(=O)N2)C
COCCNc1c(nc2n1CC[NH2+]C2)c3ccc(c(c3)OC)OC
Cc1[nH]c(c[nH+]1)C[NH+]2CCC(CC2)(C(=O)[O-])n3ccc(n3)C(C)C
COc1ccc(cc1)S(=O)(=O)N2CCN(CC2)C[C@@]3(CC[NH2+]C3)O
C[C@H](C(=O)NC(=O)NC12CC3CC(C1)CC(C3)C2)[NH+](C)CC(=O)N(C)C
C[C@H](C(=O)N1CCC2(CC1)CN(C(=O)O2)CC[NH+](C)C3CCCCC3)N
CCOC(=O)c1c(c([nH]c1C)C(=O)NC[C@@H](C)N2CC[NH+](CC2)C)C
Cc1[nH+]ccn1C[C@H](C)CNC(=O)N[C@H]2CC(=O)N(C2)C(C)(C)C
CC[C@@H](C)n1c(ccn1)NC(=O)[C@@H](C)NC[C@@H]2CCCn3c2[nH+]cc3
C[C@@H](C(C)(C)C[NH+](C)C)NC(=O)NC1CCN(CC1)S(=O)(=O)C
CC(C)(C)OC(=O)NCC[NH+]1CCC(CC1)[C@H](c2nccn2C)O
Cc1c(c(n(n1)C)C)CCNC(=O)NC[C@@H]2C[NH+]3CCCC[C@@H]3CO2
C[C@H](CNC(=O)NC[C@@H]1CN2CCCC[C@@H]2CO1)C[NH+]3CCN(CC3)C
C[C@@H](CNC(=O)NC[C@H]1C[NH+]2CCCC[C@@H]2CO1)CN3CCOCC3
Cn1cc[nH+]c1SCCNC(=O)[C@@H]2CC(=O)Nc3c2cc(cc3)OC
C[NH+](C)[C@@H]1CC[C@H](C1)NC(=O)NC2CCN(CC2)S(=O)(=O)C3CC3
C[C@H](C(=O)NC1CC1)N2CCN(CC2)C(=O)N[C@H]3CC[C@@H](C3)[NH+](C)C
C[NH+](C)[C@@H]1CC[C@H](C1)NS(=O)(=O)c2cc([nH]c2)C(=O)N3CCCC3
c1cn(cn1)CCNC(=O)NCC2(CCCCC2)C[NH+]3CCOCC3
CC[C@@H](CO)[NH+]1CCN(CC1)S(=O)(=O)c2cnc([nH]2)C(C)C
C=CCNS(=O)(=O)c1ccc(cc1)C(=O)N2CCC[C@@H]2c3[nH]cc[nH+]3
Cc1cc[nH+]c(c1)NCCNC(=O)C2CCN(CC2)C(=O)COC
c1c(c[nH]c1C#N)C(=O)N2CC[NH+](CC2)CCNC(=O)C3CCCC3
CC(C)Nc1c(cc2c([nH+]1)CCN(CC2)Cc3cnn(c3)C)C(=O)NC
CCc1nnc(o1)[C@@H](C)[NH2+][C@H](C)c2cccc(c2)S(=O)(=O)NC
c1ccc(c(c1)N)NC(=O)c2ccc(cc2)CNc3nccc(n3)c4cccnc4
CCOc1cc(ccc1O)[C@H]2C(=C(NC(=S)N2)C)C(=O)Nc3ccccc3C
c1cc(sc1)C(=O)[C@H]2[C@H](NC(=O)N[C@@]2(C(F)(F)F)O)c3ccc(cc3)O
c1cc(oc1C(=O)Nc2c(nc([nH]c2=O)SC3CCCC3)N)Br
Cc1cc(c(c(=O)[nH]1)[C@H](c2cccc(c2)OC)c3c(cc(nc3O)C)O)O
Cc1ccc(c(c1)Br)OCC(=O)NC(=S)Nc2ccccc2C(=O)N
C[C@@H](C(=O)NC(=O)NC1CC1)Sc2nc(c3c4c(sc3n2)CCCC4)N
Cc1cccc(c1OC)C(=O)NC(=S)Nc2c(c3c(s2)CCCC3)C(=O)N
c1cc2c(cc1C(=O)NC[C@H]3CCCO3)[C@@H]4C=CC[C@H]4[C@@H](N2)c5ccc(cc5O)O

c1ccc(c(c1)COc2ccc(cc2)[C@H]3CC(=O)Nc4c3c(=O)[nH]c(n4)N)Cl
COc1cccc(c1)NC(=O)[C@H]2[C@@H](C(=C(N=C2[S-])[NH3+])C#N)c3ccc(cc3)F
CC(C)(CCc1c(ccc(c1OC)[C@H]2COc3cc(cc(c3C2=O)O)O)O)O
CCCCCC(=O)O[C@@H]1C=C([C@]([C@@]2([C@H]1C(CC[C@@H]2O)(C)C)C)(CO)O)CO
CC(C)[C@@H](C(=O)NNC(=S)NC1CCCCC1)NC(=O)c2c(cccc2F)F
C[C@@H](C(=O)Nc1cc(ccc1Cl)C(F)(F)F)Sc2nc([nH]n2)N
C[C@H]1CC(=O)[C@@H]([C@@H]2[C@@H]1C[C@@H](C(=C2)C)O[C@@H]3[C@@H]([C@@H]([C@@H]([C@@H](O3)CO)O)O)O)C(C)C
C[C@H]([C@@H]1C[C@@H]([C@@]2(N1)c3ccccc3NC2=O)C(=O)Nc4ccc(cc4)OC)O
CN([C@@H](c1c(cc(cc1O)O)O)C(=O)NC2CCCCC2)C(=O)CCl
c1ccc\2c(c1)CC3(CCCC3)N/C2=C\C(=O)C(=O)N/N=C/c4ccc(c(c4)O)O
c1ccc(c(c1)S(=O)(=O)NC2(CCCC2)/C(=N\O)/N)Br
c1cc(c(cc1Br)F)Nc2c(c(ncn2)Nc3ccncc3)N
c1cc(c(cc1Nc2c(c(ncn2)NCCN3CCCCC3)N)Cl)F
c1cc(c(cc1Cl)[N-]S(=O)(=O)c2cc(c[nH+]c2NN)Br)F
CCc1cccc(c1NC(=O)N[C@@H](C)c2ccc(cc2)NC(=O)NC3CC3)C
Cc1c(cccc1NC(=O)C(=O)N[C@H](C)c2ccc(c(c2)Cl)Cl)C(=O)N
COc1ccc(cc1Cl)NC(=O)c2cc(cnc2NN)Br
c1ccc2c(c1)c(c[nH]2)C[C@@H]3C(=O)N[C@H](C(=O)N3)Cc4c[nH]c5c4cccc5
C[C@H]1CCCC[C@@H]1NC(=O)NC(=O)CNc2ccc(c(c2)Cl)C(=O)NC
C[C@@H]1CCCC[C@H]1NC(=O)NC(=O)CNc2ccc(c(c2)Cl)C(=O)NC
CCOc1ccc(cc1NC(=O)C[C@@H](c2cccs2)NC(=O)N)Cl
c1cc(oc1)C(=O)Nc2ccc(s2)C(=O)Nc3cc(c(c(c3)Cl)N)Cl
CCCNC(=O)[C@H](C)NS(=O)(=O)c1cc(cc(c1Br)N)Cl
CC[C@@H](C(=O)N)N1CCc2c(cccc2NC(=O)Nc3ccc(cc3)C)C1
CCc1ccc(cc1S(=O)(=O)N)NC(=O)Cc2c[nH]c3c2ccc(c3)Cl
C[C@@H](C(=O)Nc1ncc(s1)Cc2ccc(cc2Cl)Cl)NC(=O)N
CC(C)[NH+](C)c1cccc(c1)NC(=O)NC[C@H](COc2ccc(c(c2)F)F)O
C[C@@H](Cc1cccs1)NS(=O)(=O)c2cc(cnc2NN)Br
CC(C)[NH+](C)c1cccc(c1)NC(=O)C(=O)NCCC2CC[NH+](CC2)C
C[C@H](c1ccc(cc1)Cl)NC(=O)Nc2ccc(cc2)OCCC(=O)N
C[C@@H](C(=O)Nc1cccc(c1)C(F)(F)F)Nc2ccc3c(c2)[nH]c(=O)[nH]3
c1cc(c(c(c1)Cl)Cl)[C@H](C#N)NC(=O)C[C@H](c2cccs2)NC(=O)N
c1cc([nH]c1)[C@H]2CCCN2CC(=O)Nc3c(c4c(s3)CCCC4)C(=O)N
Cc1cccc(c1NCC(=O)N[C@H](c2ccc(cc2)OC)C(C)C)C(=O)N
CCCc1c2c([nH]n1)OC(=C([C@@H]2c3c[nH]nc3c4ccc(cc4)F)C#N)N
Cc1c(cccc1F)c2ccc(o2)CNc3ccc4c(c3)c([nH]n4)C(=O)N
CC(C)[C@@H](/C(=N/O)/N)NS(=O)(=O)c1cc(ccc1Br)Br
c1ccc2c(c1)c(c[nH]2)C[C@H](CO)NC(=O)Nc3ccc(c(c3)Cl)C#N
CC(C)[C@@H](C(=O)NNC(=O)NC)Nc1ccc(cc1Cl)C(F)(F)F
CCOc1ccc(cc1)c2c3c([nH]n2)OC(=C([C@H]3c4ccc(cc4)O)C#N)N
c1ccc2c(c1)c(c[nH]2)CC(=O)NNC(=O)N[C@H]3CCc4c3ccc(c4)Cl
Cc1ccc(cc1)c2cc(n[nH]2)C(=O)NNC(=O)N[C@@H]3CCc4c3cccc4F
c1cc(nc2c1cc(cc2)F)C[NH2+]C3[C@H]4[C@@H]3CN(C4)c5ncc(cn5)C(=O)NO
c1csc2c1c(=O)[nH]c(n2)C[NH+](CCO)Cc3[nH]c(=O)c4ccsc4n3
c1cc(ccc1C(F)(F)F)S(=O)(=O)NNC(=O)[C@]2(C[NH+]3CCC2CC3)O
c1cc(sc1)[C@@H]2CCC[NH+]2CC(=O)c3c(n(c(=O)[nH]c3=O)C4CC4)N
c1ccc(c(c1)N2C(=O)[C@H](C(=NC2=O)O)/C=N/[C@@H]3CCCC[C@@H]3[NH3+])Cl
CCOc1ccccc1N2C(=O)[C@@H](C(=NC2=O)O)/C=N/[C@@H]3CCCC[C@@H]3[NH3+]
c1ccc2c(c1)[C@](C(=O)N2C[NH+]3CCCCC3)([C@@H]4C(=O)N=C(S4)N)O
c1nc2c3c4c(c(nc3sc2c(n1)NCC[NH3+])N5CCOCC5)CCC4
C[NH+]1CCN(CC1)[C@H]2[C@H](O[C@H]([C@H]2O)CO)CNS(=O)(=O)c3ccc(cc3)Cl
c1csc(c1C(=O)N)NC(=O)CN2CC[NH+](CC2)CC(=O)N3CCCCCC3
COc1ccc(c(c1)[C@H]2[C@@H]3C[NH2+]CC=C3C(=C(C2(C#N)C#N)N)C#N)OC

C1Cc2c(sc(c2C(=O)N)NC(=O)CN3CC[NH+](CC3)[C@@H]4CCS(=O)(=O)C4)C1
COc1ccc(cc1Br)[C@H]2c3c(c(=O)cc(o3)CO)OC(=C2C#N)[NH3+]
CC[NH+]1CCN(CC1)C(=O)c2c(c\3c(o2)CCC/C3=N\NC(=S)N)C
C[NH+]1CCCN(CC1)S(=O)(=O)c2cc(cnc2NN)Br
CN(Cc1cc[nH+]cc1)S(=O)(=O)c2cc(cnc2NN)Br
C[C@@H](c1ccc(cc1)OC(C)C)[NH2+][C@H](C)C(=O)c2c(n(c(=O)n(c2=O)C)C)N
C[C@H](c1ccccc1Cl)[NH2+][C@H](C)C(=O)c2c(n(c(=O)n(c2=O)C)C)N
Cn1cc[nH+]c1CN(C)S(=O)(=O)c2cc(cnc2NN)Br
c1ccc2=[NH+][C@@H](C=c2c1)C(=O)NCCNC(=O)C(=O)NC3CCCCCC3
Cn1c(nnc1SCC(=O)NC[C@H]2CCCO2)c3c(c4ccc[nH+]c4s3)N
CN1[C@H](C(=C(N(C1=O)C)[O-])C(=O)C[NH+]2CCC(CC2)C3=c4ccccc4=[NH+]C3)N
CC[NH+]1C[C@@H]2[C@H](C1)[C@](N[C@@H]2c3cc4c(c(c3)OC)OCO4)(CCO)CO
CCC[NH+]1CC[C@@H](C1)CNC(=O)C(=O)Nc2cc(ccc2Cl)NC(=O)C
COc1cccc(c1)CN2CCC[C@@](C2=O)(C[NH+]3CCc4c(sc(n4)N)C3)O
COc1cccc(c1OC)CN2CCC[C@@](C2=O)(CN3CCC(CC3)C[NH3+])O
c1cc(c(cc1c2c3c(on2)CCN(C3)C(=O)[C@H](Cc4c[nH+]c[nH]4)N)F)F
c1cc(ccc1[C@@H]2CC(=O)Nc3c2[nH+]cn3c4ccc(cc4)F)OCC(=O)N
Cc1ccc(cc1)S(=O)(=O)NCCCNc2c3c(ncn2)C[NH2+]CC3
C[C@@H](C(=O)NCc1[nH]c2ccccc2[nH+]1)NS(=O)(=O)c3cccs3
CC(=O)N[C@H](Cc1c[nH]c2c1cccc2)C(=O)NCc3ccccc3n4cc[nH+]c4
Cc1ccc(o1)CNS(=O)(=O)c2ccc3c(c2)CN(CC3)C(=O)C(C)(C)[NH3+]
CC(C)[NH+](CCNS(=O)(=O)c1ccc2c(c1)c(=O)[nH]c(=O)[nH]2)C3CC3
CC(C)Cc1nc(on1)[C@H]2CCC[NH+](C2)Cc3ccc(c(=O)[nH]3)C(=O)N
c1cscc1CC(=O)NNC(=O)NC2CC[NH+](CC2)C[C@@H]3CCOC3
CCCCn1c(c(c(=O)[nH]c1=O)N(C)Cc2ccc(cc2)n3cc[nH+]c3)N
Cc1cc(n(n1)CC(=O)NCc2cc3n(n2)CCC[NH+](C3)CC(C)C)N
Cn1c(=O)c(c[nH]c1=O)CC[NH+]2CCC3(CC2)C(=O)Nc4ccccc4N3
CCc1nc2n(n1)CCC[C@H]2NC(=O)NC[C@H](C[NH+]3CCC(CC3)C)O
CSCc1[nH+]c2ccccc2n1CC(=O)N[C@@]3(CCCOC3)C(=O)N
CCNC(=O)[C@@H]1C[C@H](CN1Cc2c[nH]c[nH+]2)NC(=O)C3CCCCCC3
Cc1cc2c(c(c1)Cl)NC(=O)[C@@]23[C@@H]4[C@@H]([C@@H]([NH2+]3)[C@@H](C)O)C(=O)N(C4=O)CCOC
Cc1cc2c(ncnc2s1)N3CCC(CC3)[NH2+][C@@H](C)c4[nH]c(=O)[nH]n4
c1ccc2c(c1)c(c[nH]2)CC(=O)N3CCC[C@@H](C3)c4[nH+]ccn4CC(=O)N
c1cnccc1Cn2cc[nH+]c2[C@@H]3CCCN(C3)c4cc(nc(n4)N)NC5CC5
COCC(=O)N1CCc2c([nH]cn2)C13CCN(CC3)C(=O)[C@@H](C4CCCCC4)[NH3+]
Cc1cccc(c1)N2C(=O)/C(=C/NCCN3CC[NH2+]CC3)/C(=O)NC2=S
Cc1c([nH]c(n1)c2ccccc2)C(=O)N3CCNC(=O)[C@H]3Cc4c[nH+]c[nH]4
Cc1cc(c(c(c1)C)NC(=O)CNC(=O)C[NH+]2CCC[C@@H]2C(=O)NC)C
CCc1ccc([nH]1)C(=O)N2CCC3(CCN(CC3)Cc4[nH]cc[nH+]4)[C@H](C2)CO
CCn1c(c(c(=O)[nH]c1=O)C(=O)C[NH+]2CCC(CC2)Oc3ccccc3)N
c1cc(nc2c1cc(cc2)F)C[NH2+]C3[C@H]4[C@@H]3CN(C4)c5ncc(cn5)C(=O)N[O-]
Cc1cc(ccc1NS(=O)(=O)c2c[nH]c(=O)[nH]c2=O)Br
Cn1c(nnc1SCC(=O)Nc2c(c3c(s2)CCC3)C(=O)N)C(F)(F)F
COc1ccc(cc1O)/C=N/N2CC(=O)C(=C2N)c3nc4ccccc4s3
COc1cc(cc(c1O)OC)C[C@@H]2[C@H](c3ccccc3S(=O)(=O)N2)O
c1cc(ccc1/C=C/2\C(=O)NC(=S)N(C2=O)c3ccc(cc3)Cl)OCC(=O)N
CC1([C@@]([C@@](O[C@@]1(C)n2cc(c(=O)[nH]c2=O)/C=C/Br)(C)CO)(C)O)C
CS(=O)(=O)N1CCC[C@H](C1)C(=O)Nc2c(c3c(s2)CCCCC3)C(=O)N
COc1ccc(cc1OC)c2csc(n2)C3=C(N(CC3=O)c4ccc(cc4)O)N
c1cc2ccc[nH+]c2c(c1)[N-]S(=O)(=O)c3cccc(c3)S(=O)(=O)N
Cn1cccc1c2cc([nH]n2)C(=O)N3CCC[C@H]3c4ccc(s4)C(=O)N
c1cc(ccc1C[C@@H]2C(=O)N[C@H](CS2)C(=O)Nc3ccc4c(c3)NC(=O)CO4)F
Cc1c(c(=O)[nH]c(=O)[nH]1)S(=O)(=O)NCc2ccc(cc2Cl)Cl

C[C@@H](C(=O)Nc1ccc(cc1)C(F)(F)F)NC(=O)N[C@@H]2CCS(=O)(=O)C2
C[C@@H]1CN(CC(O1)(C)C)S(=O)(=O)c2cc(cnc2NN)Br
Cc1cc2c(o1)CC(C[C@@H]2NC(=O)C(=O)Nc3cccc(c3C)C(=O)NC)(C)C
CCN(C[C@H](C)C#N)S(=O)(=O)c1cc(cnc1NN)Br
C[C@@H]1CC[C@H](CN1C(=O)c2ccc3c(c2)[nH]c(=S)n(c3=O)CC=C)C(=O)N
CCN1CCN(CC1)c2ccc(cc2NC(=O)NCCNC(=O)C)Cl
Cc1cc(n(n1)CC(=O)NNC(=O)NCc2cc(cs2)Br)C
c1cc(sc1)NC(=O)N2CCC[C@H](C2)C(=O)N3CCC(CC3)C(=O)N
Cc1ccc(cc1N2CCCC2=O)NC(=O)C(=O)NCC3(CCCC3)O
C[C@@H](Cn1ccnc1)NS(=O)(=O)c2cc(cnc2N)Br
[H]/N=C\1/N(C(=O)/C(=C/c2ccccc2OC)/S1)c3ccc(cc3)S(=O)(=O)N
C[C@H](c1cccc(c1)S(=O)(=O)N)NC(=O)c2cccc(c2)N3CCCC3=O
CC[NH+](c1ccc(cc1)C(=O)N2CCC3(CC2)C(=O)NC(=O)N3)C(C)C
COc1cc(cc(c1O)OC)[C@H]2[C@@H]3C(=c4ccccc4=[NH+]3)C[C@@H](N2)C(=O)[O-]
Cc1cc(c(cc1Cn2cncn2)[C@H]3CC(=O)NCc4c3sc(n4)N)C
Cc1c(c(c(c[nH+]1)CO)CN2CC[C@H]([C@@H](C2)O)c3ccc4c(c3)OCO4)[O-]
C=CCN(C[C@H]1N[C@@H](NO1)c2ccccc2)C(=O)c3cc(c(=O)[nH]c3)Cl
COc1ccccc1[C@@H](CNC(=O)Nc2ccccc2N3CCCC3=O)O
Cc1cccc(c1)c2n[nH]c(=S)n2CCC(=O)N3C[C@@H](CC[C@@H]3C)C(=O)N
c1ccc2c(c1)[C@@]3(CCN(C3)c4ccc5cc[nH]c5n4)C(=O)N2CC(=O)N
CC(C)n1cc(c(=O)c(c1)C(=O)[O-])C(=O)N[C@H]2CC[C@H]2[NH2+]C3CCCC3
C[C@@H](C(=O)NC1CCC1)N2CC[C@]3(CCC2=O)Nc4ccccc4C(=O)N3
C[C@H](CCNS(=O)(=O)c1ccc2c(c1)CN(CC2)C(=O)c3ccc[nH]3)O
CN1C[C@@H](CCC1=O)NS(=O)(=O)c2cc(cnc2N)Br
C[C@@](CO)(C1CCCCC1)NC(=O)NC2CCN(CC2)CC(=O)N(C)C
Cc1nc2c(c(n1)C(F)(F)F)C[C@@H](CC2)C(=O)N[C@@H](COC)C(=O)N
C[C@@H](C(=O)N1CCc2c(ccs2)C1)N(C)CCCc3c(c([nH]n3)N)C#N
C[C@H](C(=O)N1CCc2c(ccs2)C1)N(C)CCCc3c(c([nH]n3)N)C#N
CC(=O)Nc1cc(cc(c1)NC(=O)C)C(=O)Nc2nc3c(s2)CCCC3
c1c(c(=O)[nH]c(n1)C23CC4CC(C2)CC(C4)C3)C(=O)N5CCC[C@H]5C(=O)N
CC(=O)Nc1ccc(c(c1)/N=C(/C(=O)N[C@H]2CC[C@@H](C2)[NH+](C)C)\[O-])Cl
C1CS(=O)(=O)C[C@H]1NC(=O)NNC(=O)CC23CC4CC(C2)CC(C4)C3
CCNc1c(cc(cn1)Br)S(=O)(=O)NC[C@@H]2CCC(=O)N2
CN(C)C[C@H]1C[C@@H](CN1S(=O)(=O)c2cc(cnc2N)Br)O
CS(=O)(=O)N[C@H]1CCCC[C@@H]1NC(=O)c2ccc3c(c2)CCC(=O)N3
C[C@H](C(=O)NC1CC1)N2CCN(CC2)C(=O)c3cc(cc4c3[nH]c(=S)[nH]4)F
CNC(=O)NNC(=O)[C@H]1CCCN(C1)C(=O)c2ccc(c(c2)Cl)Cl
CC(C)OC(=O)C1=C(Oc2c(=O)cc(oc2[C@@H]1c3ccc(c(c3)F)F)CO)N
c1ccc(cc1)CCCC(=O)[O-]
CC(=C)[C@@]1(C[C@@H]1C(=O)[O-])C
COc1cnc(nc1[S-])SC
CCCc1nnc(n1CC)[S-]
CCOc1ccc(cc1OCC)[S-]
Cc1nnc(n1[C@H](C)C(C)C)[S-]
CSc1ncc(c(n1)[O-])CC=C
c1cc(sc1)Cn2cnnc2[S-]
C1[C@H]([C@@H]1C(F)(F)F)C(=O)[O-]
CCN(C)c1nnc(s1)[S-]
CC(C)[C@@]1(C[C@@H]1C(=O)[O-])C
CCCc1nnc(n1[C@@H]2C[C@H]2C)[S-]
CCC(C)(C)c1nnc(o1)[S-]
CCC(CC)[C@H]1CC(=CC(=O)C1)[O-]
CC#CCN1CCC[C@](C1)(C)C(=O)[O-]

CC(C)c1cc(nc(n1)SC)[O-]
CCOC(=O)C(=C)C[S-]
CC(C)(C)[C@H]1[C@H](CCO1)C(=O)[O-]
C1CCC(C1)C2(CC2)C(=O)[O-]
c1c(n[n-]c1C(F)(F)F)CC#N
CSc1ccc(c(c1)[O-])C=O
c1c(cc(cc1C(F)(F)F)[O-])C=O
C1C[C@@H](C/C(=C\C(=O)[O-])/C1)C2CC2
Cc1c(sc(c1[O-])C(=O)OC)C
CC(C)C[C@@H]([C@H]1CCOC1)C(=O)[O-]
C[C@H]1CCCC[C@H]1O[C@@H](C)C(=O)[O-]
C/C(=C(/C(=O)OC)\C(F)(F)F)/[O-]
CC(C)(C)SCc1nccc(n1)[O-]
CC[C@@H](C)SC1(CCC1)C(=O)[O-]
CC[C@H]1CC[C@H](C1)c2nc(on2)[S-]
CS/C(=N\C#N)/[N-]C1CCCC1
CCCN(C(C)C)C(=O)C[S-]
C[C@H]1CCC[C@@H](C1)OC2CC(C2)[O-]
C[C@H]1CC[C@@H](C[C@@H]1C)OC2CC(C2)[O-]
CCn1c(cc(n1)C)CC2(CCC2)[O-]
CC[C@H]1CC[C@@](C1)(C#N)C2(CCC2)[O-]
CC(C)(C)S[C@H](C(=O)[O-])F
C=CC[C@]1(C[C@H]2CC[C@@H]1C2)C(=O)[O-]
CC[C@H]1CC[C@@](C1)(CCC#C)C(=O)[O-]
C/C(=C(/C(=O)[O-])\F)/CC(C)(C)OC
C/C(=C\[C@H]1CCCOC1)/C(=O)[O-]
CCOc1cc(c(s1)OCC)[O-]
C[C@H]1CCCC[C@@H]1N(C)/C=C/C(=O)[O-]
Cc1cnc(nc1)SCC[S-]
c1c(nc(nc1[O-])CCl)C(F)F
CC#CC[C@@H]1CCC[C@H]1C(=O)[O-]
CC(C)SC(C(=O)[O-])(F)F
c1cc(cc(c1)[S-])OC2CC2
CC(C)(C)Oc1ccc(c(c1)C#N)[O-]
CCCC[C@H](C(=O)OC)[S-]
CCc1cc(c(nc1C)OC)[O-]
CN1C[C@@H](CCC1=O)NS(=O)(=O)c2cc(cnc2N)Br
C[C@@](CO)(C1CCCCC1)NC(=O)NC2CCN(CC2)CC(=O)N(C)C
Cc1nc2c(c(n1)C(F)(F)F)C[C@@H](CC2)C(=O)N[C@@H](COC)C(=O)N
C[C@@H](C(=O)N1CCc2c(ccs2)C1)N(C)CCCc3c(c([nH]n3)N)C#N
C[C@H](C(=O)N1CCc2c(ccs2)C1)N(C)CCCc3c(c([nH]n3)N)C#N
CC(=O)Nc1cc(cc(c1)NC(=O)C)C(=O)Nc2nc3c(s2)CCCC3
c1c(c(=O)[nH]c(n1)C23CC4CC(C2)CC(C4)C3)C(=O)N5CCC[C@H]5C(=O)N
CC(=O)Nc1ccc(c(c1)/N=C(/C(=O)N[C@H]2CC[C@@H](C2)[NH+](C)C)\[O-])Cl
C1CS(=O)(=O)C[C@H]1NC(=O)NNC(=O)CC23CC4CC(C2)CC(C4)C3
CCNc1c(cc(cn1)Br)S(=O)(=O)NC[C@@H]2CCC(=O)N2
CN(C)C[C@H]1C[C@@H](CN1S(=O)(=O)c2cc(cnc2N)Br)O
CS(=O)(=O)N[C@H]1CCCC[C@@H]1NC(=O)c2ccc3c(c2)CCC(=O)N3
C[C@H](C(=O)NC1CC1)N2CCN(CC2)C(=O)c3cc(cc4c3[nH]c(=S)[nH]4)F
CNC(=O)NNC(=O)[C@H]1CCCN(C1)C(=O)c2ccc(c(c2)Cl)Cl
CC(C)OC(=O)C1=C(Oc2c(=O)cc(oc2[C@@H]1c3ccc(c(c3)F)F)CO)N
Cc1nc2c(c(n1)C(F)(F)F)C[C@@H](CC2)C(=O)N[C@@H](COC)C(=O)N
C[C@@H](C(=O)N1CCc2c(ccs2)C1)N(C)CCCc3c(c([nH]n3)N)C#N

C[C@H](C(=O)N1CCc2c(ccs2)C1)N(C)CCCc3c(c([nH]n3)N)C#N

Table S5. List of inactive compounds retrieved from ChEMBL.

Inactive compound SMILES (Simplified Molecular Input Line Entry System)
COc1cc(OC)c2c(N3CCCCC3)nc(-c3cc(C)c(OCCCC(=O)NO)c(C)c3)nc2c1
CCCC(CCC)C(=O)O
Nc1ccc(F)cc1NC(=O)c1ccc(CNC(=O)/C=C/c2cccnc2)cc1
Nc1ccccc1NC(=O)Cc1ccc(NC(=O)c2n[nH]cc2NC(=O)c2c(Cl)cccc2Cl)cc1
Nc1ccccc1NC(=O)c1ccc(CNC(=O)c2n[nH]cc2NC(=O)c2c(Cl)cccc2Cl)cc1
COC(=O)N(O)CCCCCCC(=O)Nc1ccccc1
CNC(=O)/C(CCCCCCC(=O)Nc1ccc(-c2ccccc2)cc1)=N\O
Nc1ccccc1NC(=O)c1ccc(CNC(=O)OCc2cccnc2)cc1
CC(C)C[C@H](NC(=O)[C@H](Cc1ccccc1)NC(=O)c1ccc(C(=O)Nc2ccccc2N)cc1)B1O[C@@H]2[C@@H]3C[C@H](C[C@]2(C)O1)C3(C)C
Nc1ccccc1NC(=O)c1ccc(C(=O)Nc2cccc(Nc3ncc(-c4cccnc4)s3)c2)s1
O=C(NO)c1cccc2ccccc12
O=C(NO)c1cccc(-c2ccccc2)c1
O=C1N[C@@H]2CCOCCSSCCCCC[C@@H]1NC(=O)[C@H]1CCCN1C(=O)[C@H](Cc1ccccc1)NC2=O
CNC(=O)/C(CCCCCCC(=O)Nc1cccc(Br)c1)=N\O
CNC(=O)/C(CCCCCCC(=O)Nc1cccc(-c2cncc3cc(Cl)ccc23)c1)=N\O
CC(=O)Nc1ccc(C(O)CSc2nc(C)cc(O)n2)cc1
COC(=O)CSc1nc(C)cc(O)n1
Nc1ccccc1NC(=O)c1ccc(Cn2cc(-c3ccc(C(=O)NCc4cccnc4)cc3)nn2)cc1
CNC(=O)/C(CCCCCCC(=O)Nc1cnc2ccccc2c1)=N\O
CNC(=O)/C(CCCCCCC(=O)Nc1cccc(-c2cccnc2)c1)=N\O
CNC(=O)/C(CCCCCCNC(=O)c1cc(-c2ccc(N)cc2)on1)=N/O
Nc1nc(Nc2ccc(F)c(Cl)c2)c2ccn(Cc3ccc(C(=O)Nc4ccccc4N)cc3)c2n1
CC(=O)SCCCCC[C@@H]1NC(=O)[C@H]2CCCN2C(=O)[C@H](Cc2ccccc2)NC(=O)[C@@H](CCOCCSC(C)=O)NC1=O
Nc1ccccc1NC(=O)c1ccc(Cn2cnc3c(Nc4ccc(F)c(Cl)c4)nc(Cl)nc32)cc1
Nc1ccccc1NC(=O)c1ccc(CNCOC(=O)c2cccnc2)cc1
Cc1ccc(NC(=O)c2ccc(C(=O)Nc3ccccc3N)nc2)cc1Nc1ncc(-c2cccnc2)cn1
Cc1ccc(NC(=O)c2ccc(C(=O)Nc3ccccc3N)s2)cc1Nc1ncc(-c2cccnc2)s1
Nc1ccccc1NC(=O)c1ccc(-c2cn(CCc3cccc([N+](=O)[O-])c3)nn2)s1
O=C(CCCCCCNC(=O)C(O)(O)C(F)(F)F)Nc1ccc(Br)cc1
COc1cc(C)nc(SCC(=O)c2ccc(NC(C)=O)cc2)n1
Cc1ccc(NC(=O)c2ccc(C(=O)Nc3ccccc3N)s2)cc1Nc1ncc(-c2cccnc2)cn1
CN1C(=O)C(NC(=O)CCCCC(=O)NO)N=C(c2ccccc2)c2ccccc21
COc1ccc(C(=O)N[C@@H](Cc2c[nH]c3ccccc23)C(=O)NCc2ccc(C(=O)NNc3ccccc3)cc2)cc1
Cc1ccc(NC(=O)c2ccc(C(=O)Nc3ccccc3N)cc2)cc1Nc1ncc(-c2cccnc2)cn1
CC(=O)CSc1nc(C)cc(O)n1
CC(=O)Nc1ccc(C(=O)CSc2cccc(O)c2)cc1
CC(=O)Nc1ccc(C(=O)COc2cc(C)cc(O)n2)cc1
CC(=O)N(C)c1ccc(C(=O)CSc2nc(C)cc(O)n2)cc1
Cc1cc(O)nc(SCC(=O)c2ccc(C(C)(C)C)cc2)n1
Cc1cc(O)nc(SCC(=O)NCCCCc2ccccc2)n1
CN(C(=O)c1ccc(C(=O)Nc2ccccc2N)cc1)c1cccc(Nc2nccc(-c3cccnc3)n2)c1
CCCNNC(=O)/C=C/c1ccc(OC[C@H](Cc2c[nH]c3ccccc23)NC(=O)c2ccc(OC)cc2)cc1
CCCNNC(=O)CCCCCNC(=O)[C@H](Cc1c[nH]c2ccccc12)NC(=O)c1ccc(OC)cc1
Nc1ccccc1NC(=O)c1ccc(CNC2=N[C@@H](c3ccccc3)[C@H](c3ccccc3)O2)cc1
Nc1ccccc1NC(=O)c1ccc(-c2cn(CCc3cccc([N+](=O)[O-])c3)nn2)s1
COc1ccc(C(=O)N[C@@H](Cc2c[nH]c3ccccc23)C(=O)NCCCCCC(=O)Nc2cc(-c3cccs3)ccc2N)cc1

Cc1cc(O)nc(SCC(=O)c2ccc3c(c2)OCCO3)n1
CCCNNC(=O)c1ccc(CNC(=O)[C@H](Cc2c[nH]c3ccccc23)NC(=O)c2ccc(OC)cc2)cc1
CC(=O)Nc1ccc(C(=O)CSc2nccc(C)n2)cc1
Cc1cc(O)nc(SCC(=O)NCCCCCCc2ccccc2)n1
CNC(=O)/C(CCCCCCC(=O)Nc1ccccc1)=N\O
Nc1ccccc1NC(=O)c1ccc(C(=O)Nc2cccc(Nc3ncc(-c4cccnc4)s3)c2)cn1
CC(=O)N(O)CCCCCC(=O)Nc1ccccc1
CC(=O)N(O)CCCCCCCC(=O)Nc1ccccc1
CC(=O)Nc1ccc(CSc2nc(C)cc(O)n2)cc1
CCCCNNC(=O)c1ccc(CNC(=O)[C@H](Cc2c[nH]c3ccccc23)NC(=O)c2ccc(OC)cc2)cc1
COc1ccc(C(=O)N[C@@H](Cc2c[nH]c3ccccc23)C(=O)NCc2ccc(C(=O)Nc3ccccc3N)cc2)cc1
CCCNNC(=O)CCCCCCCNC(=O)[C@H](Cc1c[nH]c2ccccc12)NC(=O)c1ccc(OC)cc1
CNC(=O)/C(CCCCCCNC(=O)c1cc(-c2cccc(NC(=O)OC(C)(C)C)c2)on1)=N/O
CNC(=O)/C(CCCCCCC(=O)Nc1ccc(N(C)C)cc1)=N\O
CNC(=O)/C(CCCCCCC(=O)Nc1cccc(-c2ccc(C(=O)O)cc2)c1)=N\O
COc1ccc(C(=O)N[C@@H](Cc2c[nH]c3ccccc23)C(=O)NCCCCCC(=O)Nc2ccc(F)cc2N)cc1
CC1(C)C(=O)N(c2ccc(C#N)c(C(F)(F)F)c2)C(=S)N1c1ccc(C(=O)NCCCCCCn2cccc(O)c2=O)c(F)c1
CCCCCNNC(=O)c1ccc(CNC(=O)[C@H](Cc2c[nH]c3ccccc23)NC(=O)c2ccc(OC)cc2)cc1
COc1ccc(C(=O)N[C@@H](Cc2c[nH]c3ccccc23)C(=O)NCc2ccc(C(=O)NNC3CCCC3)cc2)cc1
CC(=O)Nc1ccc(C(=O)CSc2nc(C)cc(C)n2)cc1
CN1C(=O)CN=C(c2ccccc2)c2cc(C#CCCC(=O)NO)ccc21
CNC(=O)/C(CCCCCCC(=O)Nc1ccc2ccccc2c1)=N\O
Cc1ccc(NC(=O)c2ccc(C(=O)Nc3ccccc3N)nc2)cc1Nc1ncc(-c2cccnc2)s1
C/C=C1\NC(=O)[C@@H](CSC)NC(=O)[C@@H](C(C)C)CC(=O)C[C@@H](/C=C/CCSC)OC(=O)[C@H](C(C)C)NC1=O
Cc1cc(O)nc(SCC(=O)C(C)(C)C)n1
Nc1ccccc1NC(=O)c1ccc(-c2cn(CCc3cccc([N+](=O)[O-])c3)nn2)s1
CN1C(=O)C(N)N=C(c2ccccc2)c2cc(C#CCCCC(=O)NO)ccc21
CNC(=O)/C(CCCCCCC(=O)NC1c2ccccc2-c2ccccc21)=N\O
CNC(=O)/C(CCCCCCC(=O)Nc1cccc(-c2ccccc2)c1)=N\O
CNC(=O)/C(CCCCCCNC(=O)c1cc(-c2ccc(NC(=O)OC(C)(C)C)cc2)on1)=N/O
CNC(=O)/C(CCCCCCNC(=O)c1cc(-c2cccc(N)c2)on1)=N/O
Cc1cc(O)nc(SCC(=O)NCCc2ccccc2)n1
CCCNNC(=O)CCCCCCNC(=O)[C@H](Cc1c[nH]c2ccccc12)NC(=O)c1ccc(OC)cc1
Nc1ccccc1NC(=O)c1ccc(Cn2ccc3c(Cl)nc(Cl)nc32)cc1
Nc1nc(Cl)c2ccn(Cc3ccc(C(=O)Nc4cc(F)ccc4N)cc3)c2n1
O=C(O)C1CCN(CCOc2ccc3cc2COC/C=C/COCc2cccc(c2)-c2ccnc(n2)N3)CC1
COc1cc(Nc2nc(N)nc3c2ncn3Cc2ccc(C(=O)Nc3ccccc3N)cc2)cc(OC)c1
Nc1ccccc1NC(=O)c1ccc(-c2cn(CCc3ccsc3)nn2)cc1
Nc1nc(Cl)c2ncn(Cc3ccc(C(=O)Nc4ccccc4N)cc3)c2n1
Nc1nc(Nc2ccc(F)c(Cl)c2)c2ncn(Cc3ccc(C(=O)Nc4ccccc4N)cc3)c2n1
Cn1c(CCC(=O)O)nc2ccccc21
C=Cc1c(C)c2cc3nc(c(C(=O)OC)c4[nH]c(cc5nc(cc1[nH]2)C(C)=C5CC)c(C)c4C(=O)OC)[C@@H](CCC(=O)Nc1ccc(C(=O)Nc2ccccc2N)cc1)[C@@H]3C
Nc1ccccc1NC(=O)c1ccc(-c2cn(CCc3cccc([N+](=O)[O-])c3)nn2)s1
CC1(C)C(=O)N(c2ccc(C#N)c(C(F)(F)F)c2)C(=S)N1c1ccc(C(=O)NCCCNS(N)(=O)=O)c(F)c1
O=C(O)c1ccc(OCCOc2ccc3cc2COC/C=C/COCc2cccc(c2)-c2ccnc(n2)N3)cc1
Nc1ccccc1NC(=O)c1ccc(-c2cn(CCc3ccsc3)nn2)cc1
Cc1c[nH]nc1Nc1nc(Cl)nc2c1ncn2Cc1ccc(C(=O)Nc2ccccc2N)cc1
COc1ccc2sc(NC(=O)CCCCCCNC(=O)C(O)(O)C(F)(F)F)nc2c1
CCc1c(C(=O)Nc2cccc(CCC(=O)Nc3ccccc3N)c2)[nH]c(C)c1C(C)=O
Nc1ccccc1NC(=O)c1ccc(-c2cn(CCc3ccsc3)nn2)cc1
CCC(=O)CCCCC[C@@H]1NC(=O)[C@H](C)N(C)C(=O)C[C@H](CC(C)C)NC(=O)[C@H](Cc2c[nH]c3ccccc23)NC1=O
CC(=O)SCCCCCCNC(=O)c1cc(-c2ccc(F)cc2)nn1C
CC(CN(C)C)N(C)C(=O)c1ccc(-c2noc(C(F)(F)F)n2)cc1F

CC1(C)c2cccc(C(=O)NO)c2CN1c1cnc(C(F)(F)F)cn1
CC1(C)c2cccc(C(N)=O)c2CN1c1cnc(C(F)(F)F)cn1
Nc1ccccc1NC(=O)CCCNC(=O)/C(Cc1ccc(O)c(Br)c1)=N/O
CCCCn1nc(-c2ccc(F)cc2)cc1C(=O)NCCCCCCS
Nc1ccccc1NC(=O)c1ccc(CN(CC(=O)NCc2ccccc2)C(=O)c2cccnc2)cc1
CCC(C)n1nc(-c2ccc(F)cc2)cc1C(=O)NCCCCCCS
Cc1c(-c2ccc(F)cc2)nn(C)c1C(=O)NCCCCCCS
Nc1ccccc1NC(=O)c1ccc(C(C(=O)Nc2ccccc2)C(=O)Nc2ccccc2)cc1
CCC(=O)CCCCC[C@@H]1NC(=O)C[C@H](CC(C)C)NC(=O)[C@H](Cc2c[nH]c3ccccc23)NC(=O)[C@H](CCCCCC(=O)CC)NC1=O
CC(=O)NCCCC[C@@H]1NC(=O)C[C@H](CC(C)C)NC(=O)[C@H](Cc2c[nH]c3ccccc23)NC(=O)[C@H](CSCCC(=O)O)NC1=O
Cc1cc(C)cc(C(=O)N(CC(=O)NCc2ccccc2)Cc2ccc(C(=O)Nc3cc(-c4ccccc4)ccc3N)cc2)c1
O=C(NCCCCCNC(=O)c1nc(-c2nccs2)sc1C1CC1)c1cccc(-c2noc(C(F)(F)F)n2)c1
CCC(=O)CCCSC[C@@H]1NC(=O)[C@H](C)NC(=O)C[C@H](CC(C)C)NC(=O)[C@H](Cc2c[nH]c3ccccc23)NC1=O
CC(C)C[C@H]1CC(=O)N[C@@H](C)C(=O)N[C@@H](CSCC(=O)O)C(=O)N[C@@H](Cc2c[nH]c3ccccc23)C(=O)N1
N=C(N)NCCC[C@@H]1NC(=O)C[C@H](Cc2ccc(O)cc2)NC(=O)[C@H](CC(=O)O)NC(=O)[C@H](CCCCCC(=O)O)NC1=O
NS(=O)(=O)NCCCCC(=O)Nc1cccc(-c2ccccc2)c1
CNS(=O)(=O)NCCCCCC(=O)Nc1cccc(-c2ccccc2)c1
NS(=O)(=O)NCCCCCCC(=O)Nc1cccc(-c2ccccc2)c1
NS(=O)(=O)OCCCCCC(=O)Nc1cccc(-c2ccccc2)c1
CCC(=O)CCSC[C@@H]1NC(=O)[C@H](C)NC(=O)C[C@H](CC(C)C)NC(=O)[C@H](Cc2c[nH]c3ccccc23)NC1=O
CCC(=O)CCCCC[C@@H]1NC(=O)[C@H](CCCCNC(C)=O)NC(=O)CCN(CC(C)C)C(=O)CN(CCc2c[nH]c3ccccc23)C1=O
CC(C)N(C)C(=O)c1ccc(-c2noc(C(F)(F)F)n2)cc1
O=C(c1ccc(-c2noc(C(F)(F)F)n2)cc1)N1CCOCC1
CC(CNC(=O)c1ccc(-c2noc(C(F)(F)F)n2)cc1)N(C)C
CN(C)C(=O)CNC(=O)c1ccc(-c2noc(C(F)(F)F)n2)cc1
CC(C)(C)OC(=O)N1CCN(C(=O)c2ccc(-c3noc(C(F)(F)F)n3)cc2)CC1
O=C(NO)NCCCCNC(=O)c1nc(-c2nccs2)sc1C1CC1
O=C(NO)C(c1ccccc1)c1ccccc1
CC(C)[C@@H](CN(C)C)NC(=O)c1ccc(-c2noc(C(F)(F)F)n2)cc1
CC(C)[C@H](CN(C)C)NC(=O)c1ccc(-c2noc(C(F)(F)F)n2)cc1F
CCN(C)C[C@@H](C)NC(=O)c1ccc(-c2noc(C(F)(F)F)n2)cc1
O=C(Nc1ccnc(F)c1)c1ccc(-c2noc(C(F)(F)F)n2)cc1
CN(CCO)C(=O)c1ccc(-c2noc(C(F)(F)F)n2)cc1
O=C(c1ccc(-c2noc(C(F)(F)F)n2)cc1)N1CCC(O)C1
CCN(CC)CC(C)NC(=O)c1ccc(-c2noc(C(F)(F)F)n2)cc1
CCN(CC)C[C@H](NC(=O)c1ccc(-c2noc(C(F)(F)F)n2)cc1)C(C)C
CC(=O)NS(=O)(=O)NCCCCCC(=O)Nc1cccc(-c2ccccc2)c1
O=C(NO)c1cccc2c1CCN(c1nc3ccccc3[nH]1)C2
CN1CCC(CNC(=O)c2ccc(-c3noc(C(F)(F)F)n3)cc2)CC1
O=C(Nc1ccnc(O)c1)c1ccc(-c2noc(C(F)(F)F)n2)cc1
C[C@@H](CN(C)C)NC(=O)c1ccc(-c2noc(C(F)(F)F)n2)cc1
CCC(=O)CCCCC[C@@H]1NC(=O)[C@@H]2CCCN2C(=O)C[C@H](CC(C)C)NC(=O)[C@H](Cc2c[nH]c3ccccc23)NC1=O
CC(C)n1nc(C(=O)NCCCCCCS)cc1-c1ccc(F)cc1
CCC(C)n1nc(C(=O)NCCCCCCS)cc1-c1ccc(F)cc1
CCC(=O)CCCCC[C@@H]1NC(=O)[C@H](Cc2ccccc2)NC(=O)C[C@H](CC(C)C)NC(=O)[C@H](Cc2c[nH]c3ccccc23)NC1=O
O=C(O)CCNC(=O)/C(Cc1ccc(O)c(Br)c1)=N/O
CCCNC(=O)/C(Cc1ccc(O)c(Br)c1)=N/O
CCC(=O)CCCCC[C@@H]1NC(=O)[C@@H](C)n2cc(nn2)[C@H]([C@@H](C)CC)NC(=O)[C@H](Cc2c[nH]c3ccccc23)NC1=O
Cc1cc(C)cc(C(=O)N(CCCCCC(=O)Nc2ccccc2N)CC(=O)NCc2ccccc2)c1

Cc1ccc(CNC(=O)CN(CCCCCC(=O)Nc2ccccc2N)C(=O)c2cc(C)cc(C)c2)cc1
Cc1cc(C)cc(C(=O)N(CC(=O)NCc2ccccc2)Cc2ccc(C(=O)Nc3cc(F)ccc3N)cc2)c1
O=C(CCS)NCCNC(=O)c1cc(-c2ccc(F)cc2)[nH]n1
C[C@H](CN(C)C)NC(=O)c1c(F)cc(-c2noc(C(F)(F)F)n2)cc1F
CCN(CC)C[C@H](C)NC(=O)c1ccc(-c2noc(C(F)(F)F)n2)cc1F
CCC(=O)CCCCC[C@@H]1NC(=O)[C@H](C)N(C)C(=O)C[C@H](CC(C)C)NC(=O)[C@H](Cc2c[nH]c3ccccc23)NC1=O
CC(C)CNC(=O)c1ccc(-c2noc(C(F)(F)F)n2)cc1
O=C(NC1CCCNC1)c1ccc(-c2noc(C(F)(F)F)n2)cc1
CC(C)C[C@H](CN(C)C)NC(=O)c1ccc(-c2noc(C(F)(F)F)n2)cc1
CCC(=O)CCCSC[C@@H]1NC(=O)[C@H](C)NC(=O)C[C@H](CC(C)C)NC(=O)[C@H](Cc2c[nH]c3ccccc23)NC1=O
CCC(=O)CCCCC[C@@H]1NC(=O)[C@H](CCCCCNC(C)=O)NC(=O)C[C@H](CC(C)C)NC(=O)[C@H](Cc2c[nH]c3ccccc23)NC1=O
O=C(O)CCCCC[C@@H]1NC(=O)[C@H](Cc2c(F)c(F)c(F)c(F)c2F)NC(=O)C[C@H](Cc2ccc(O)cc2)NC(=O)[C@H](Cc2cccc3ccccc23)NC1=O
CCC(=O)CCSC[C@@H]1NC(=O)[C@H](C)NC(=O)C[C@H](CC(C)C)NC(=O)[C@H](Cc2c[nH]c3ccccc23)NC1=O
COc1cc2c(NCC3CCN(C)CC3)cc(-c3ccc(C)o3)nc2cc1OCCCN1CCCC1
CC(=O)Nc1ccc(C(=O)Nc2cc(-c3cccs3)ccc2N)cc1
CCC(CC)NC(=O)c1ccc(-c2noc(C(F)(F)F)n2)cc1
CC(C)(C)OC(=O)N1CCC(NC(=O)c2ccc(-c3noc(C(F)(F)F)n3)cc2)CC1
CCN(CC)C[C@@H](C)NC(=O)c1ccc(-c2noc(C(F)(F)F)n2)nc1
CCC(=O)CCCCC[C@@H]1NC(=O)[C@H](C)NC(=O)C[C@H](CC(C)C)NC(=O)[C@H](Cc2cccc3ccccc23)NC1=O
CCCn1nc(C(=O)NCCCCCCS)cc1-c1ccc(F)cc1
C[C@H](CN(C)C)NC(=O)c1ccc(-c2noc(C(F)(F)F)n2)cc1Cl
CN(C)C1CCN(C(=O)c2ccc(-c3noc(C(F)(F)F)n3)cc2)CC1
CCCCn1nc(C(=O)NCCCCCCS)cc1-c1ccc(F)cc1
COc1ccc2c(C(=O)c3cc(OC)c(OC)c(OC)c3)cn(S(=O)(=O)c3ccc(/C=C/C(=O)NO)cc3)c2c1
CN(C)c1cccc2c(S(=O)(=O)N(CC(=O)NCc3ccccc3)Cc3ccc(C(=O)Nc4ccccc4N)cc3)cccc12
C=C1CN(Cc2ccc(C(=O)Nc3ccccc3N)cc2)C(=O)c2ccccc21
CC(C)N(C)C[C@@H](C)NC(=O)c1ccc(-c2noc(C(F)(F)F)n2)cc1
Nc1ccccc1NC(=O)c1ccc(-c2ccc3ncnc(Nc4ccc(OCc5cccc(F)c5)c(Cl)c4)c3c2)s1
CN(C)c1ccc(C(=O)N(CCCCCC(=O)Nc2ccccc2N)CC(=O)NCc2ccccc2)cc1
COc1ccc(CNC(=O)CN(CCCCCC(=O)Nc2ccccc2N)C(=O)c2cc(C)cc(C)c2)cc1
Nc1ccccc1NC(=O)CCCCNC(=O)c1nc(-c2nccs2)sc1C1CC1
O=C(NCCCCCCS)c1cc(-c2ccc(F)cc2)nn1Cc1ccccc1
O=C(NC1CCNC1)c1ccc(-c2noc(C(F)(F)F)n2)cc1
CC(CN(C)C)N(C)C(=O)c1ccc(-c2noc(C(F)(F)F)n2)cc1
O=C(Nc1ccccc1)c1ccc(-c2noc(C(F)(F)F)n2)cc1
CC[C@@H]1OC(=O)[C@@H](C)[C@H](O[C@H]2C[C@@](C)(OC)[C@@H](O)[C@H](C)O2)[C@H](C)[C@H](O[C@@H]2O[C@H](C)C[C@H](N(C)Cc3ccc(-c4cn(CC(=O)NO)nn4)cc3)[C@H]2O)[C@@](C)(OC)C[C@H](C)C(=O)[C@@H](C)[C@@H](O)[C@@]1(C)O
Cc1ccc2c(c1)nc(CCN1C(=O)c3cccc4cccc(c34)C1=O)n2-c1ccncc1
C[C@H](CN(C)C)NC(=O)c1ccc(-c2noc(C(F)(F)F)n2)cc1
CC1(C)C(=O)N(c2ccc(C#N)c(C(F)(F)F)c2)C(=S)N1c1ccc(C(=O)NCCCCCCc2ccc(C(=O)C(F)(F)F)s2)c(F)c1
Nc1ccccc1NC(=O)c1ccc(-c2ccc3ncnc(Nc4ccc(OCc5cccc(F)c5)c(Cl)c4)c3c2)o1
CCC(=O)CCCCC[C@@H]1NC(=O)[C@H](C)NC(=O)C[C@H](CC(C)C)NC(=O)[C@H](Cc2c[nH]c3ccccc23)NC1=O
CC(=O)NCCCCC[C@@H]1NC(=O)C[C@H](CC(C)C)NC(=O)[C@H](Cc2cccc3ccccc23)NC(=O)[C@H](CCCCCC(=O)O)NC1=O
CCC(=O)CCCCC[C@@H]1NC(=O)[C@H](C)NC(=O)C[C@H](CC(C)C)NC(=O)[C@H](Cc2c[nH]c3ccccc23)NC1=O
CCC(=O)CCCCC[C@@H]1NC(=O)[C@@H]2CCCN2C(=O)C[C@H](CC(C)C)NC(=O)[C@H](Cc2c[nH]c3ccccc23)NC1=O
O=S(=O)(NCC1CCCCC1)c1ccc(-c2noc(C(F)(F)F)n2)cc1
CCN(CC)C[C@H](NC(=O)c1ccc(-c2noc(C(F)(F)F)n2)cc1F)C(C)C
CCCn1nc(-c2ccc(F)cc2)cc1C(=O)NCCCCCCS
CCC(=O)CCCCC[C@@H]1NC(=O)[C@H](CCCNC(=N)N)NC(=O)CCN(CC(C)C)C(=O)CN(CCc2c[nH]c3ccccc23)C1=O
CCC(=O)CCCCC[C@@H]1NC(=O)[C@H](C)N(C)C(=O)CCN(CC(C)C)C(=O)CN(CCc2c[nH]c3ccccc23)C1=O

O=C(CCC(=O)NCCCN1c2ccccc2CCc2ccc(Cl)cc21)NO
NC(=O)C1CCCc2c(-c3ccc(Cl)c(Cl)c3)n[nH]c21
CC1(C)C(=O)N(c2ccc(C#N)c(C(F)(F)F)c2)C(=S)N1c1ccc(C(=O)NCCCCCCn2cccc(O)c2=S)c(F)c1
CC(C)C[C@H]1CC(=O)N[C@@H](C)C(=O)N[C@@H](CSCCC(=O)O)C(=O)N[C@@H](Cc2c[nH]c3ccccc23)C(=O)N1
CC1(C)C(=O)N(c2ccc(C#N)c(C(F)(F)F)c2)C(=S)N1c1ccc(C(=O)NCCCCCCC(=O)Nc2ccccc2N)c(F)c1
O=C(NCCCCC(O)C(F)(F)F)c1nc(-c2nccs2)sc1C1CC1
CCC(=O)CCCCC[C@@H]1NC(=O)[C@H](CCCNC(=N)N)NC(=O)CCN(CC=C(C)C)C(=O)CN(CCc2c[nH]c3ccccc23)C1=O
CN(CCCCCC(=O)Nc1cccc(-c2ccccc2)c1)S(N)(=O)=O
CC(=O)SCC/C=C/[C@@H]1CC(=O)N[C@H](C)C(=O)N[C@H](C)C(=O)N[C@H](C(C)C)C(=O)NCC(=O)O1
Nc1ccccc1NC(=O)CCCCCN(CC(=O)NC1CCCCC1)C(=O)c1cccc2ccccc12
CCC(=O)CCCCC[C@@H]1NC(=O)[C@H](C)NC(=O)C[C@H](CC(C)C)NC(=O)[C@H](Cc2cccc3ccccc23)NC1=O
O=C(CCCCCCC(=O)Nc1ccccc1)NOCc1ccccc1
CCn1nc(C(=O)NCCCCCCS)cc1-c1ccc(F)cc1
CCCNNC(=O)CCCCCCC(=O)Nc1ccccc1
CN(C)S(=O)(=O)NCCCCCC(=O)Nc1cccc(-c2ccccc2)c1
CC[C@@H](C)n1cc(C)c2c(C(=O)NCc3c(C)cc(C)[nH]c3=O)cc(-c3ccc(N4CCN(c5ncc(C(=O)NO)cn5)CC4)nc3)cc21
NS(=O)(=O)NC(=O)CCCCCC(=O)Nc1cccc(-c2ccccc2)c1
O=C(NCCCCCCS)c1cc(-c2ccc(F)cc2)n(Cc2ccccc2)n1
CCC(=O)CCCCC[C@H](NC(=O)[C@H]1CC12CCN(C)CC2)c1ncc(-c2ccc3nnccc3c2)[nH]1
COc1ccc(-c2ncc(/C=C/C(=O)Nc3ccccc3N)s2)c(OC)c1OC
CN(C)c1ccc(C(=O)N(CC(=O)NCc2ccccc2)Cc2ccc(C(=O)Nc3cc(-c4ccccc4)ccc3N)cc2)cc1
Cc1cc(-c2nc3sc4c(c3c(=O)[nH]2)CCN(C)C4)cc(C)c1OCc1cccc(C(=O)Nc2ccccc2N)c1
O=S(=O)(N[C@@H]1CCNC1)c1ccc(-c2noc(C(F)(F)F)n2)cc1
Nc1ccccc1NC(=O)CCNC(=O)/C(Cc1ccc(O)c(Br)c1)=N/O
Nc1ccccc1NC(=O)CCCCCN(CC(=O)NCc1ccccc1)C(=O)c1cccc2ccccc12
C/C=C1\NC(=O)c2csc(n2)[C@@H](C(C)C)NC(=O)C[C@@H](/C=C/CCSC(=O)CCCCCCC)OC(=O)[C@H](C(C)C)NC1=O
Nc1ccccc1NC(=O)c1ccc(Cn2ncc3c2nc(N)n2nc(-c4ccco4)nc32)cc1
O=C(CCCNC(=O)/C(Cc1ccc(O)c(Br)c1)=N/O)NO
O=C(O)CCCCCCCOc1ccc2cc1COC/C=C/COCc1cccc(c1)-c1ccnc(n1)N2
O=C(C[C@@H](CC1CCCC1)C(=O)N1CC2(CC2)C[C@H]1c1nc(-c2ccccc2)no1)NO
CCC(=O)CCCCC[C@@H]1NC(=O)[C@H](C)NC(=O)C[C@H](CC(C)C)NC(=O)[C@H](Cc2cccc3ccccc23)NC1=O
O=C(NO)c1csc(CCCN2CCC(CN[C@H]3C[C@@H]3c3ccccc3)CC2)n1
CN1CCC(CNS(=O)(=O)c2ccc(-c3noc(C(F)(F)F)n3)cc2)C1
Nc1ccccc1NC(=O)c1ccc(CCNc2nc(N)n3nc(-c4ccco4)nc3n2)cc1
C=CCOc1ccc2oc(C(=O)NCc3ccc(C(=O)NNCCC)cc3)cc2c1
CCCNNC(=O)c1ccc(CNC(=O)c2cc3cc(O)ccc3o2)cc1
CCCNNC(=O)c1ccc(CNC(=O)c2cc3cc(F)ccc3o2)cc1
CCCNNC(=O)c1ccc(CNC(=O)c2ccc3ccccc3c2)cc1
C[C@H](CN(C)Cc1ccccc1)NC(=O)c1ccc(-c2noc(C(F)(F)F)n2)nc1
CCCNNC(=O)/C=C/c1ccc(CNCCc2c(C)[nH]c3ccccc23)cc1
CCCNNC(=O)c1ccc(CNC(=O)/C=C/c2ccccc2)cc1
COCCN(C)C(=O)c1ccc(-c2noc(C(F)(F)F)n2)cc1
O=C(C[C@@H](CC1CCCC1)C(=O)N1CC2(CC2)C[C@H]1c1nc(-c2ccc(F)cc2C(F)(F)F)no1)NO
CCCCCCCCCCCCCCCCNNC(=O)c1cccs1
O=C(NC1CCNC1)c1ccc(-c2noc(C(F)(F)F)n2)cc1
CN(C)CC(C)(C)NC(=O)c1ccc(-c2noc(C(F)(F)F)n2)cc1
CN(C)c1ccc(C(=O)N(CCCCCC(=O)Nc2cc(F)ccc2N)CC(=O)NCc2ccccc2)cc1
Cc1cc(C)cc(C(=O)N(CCCCCC(=O)Nc2cc(-c3ccccc3)ccc2N)CC(=O)NCc2ccccc2)c1
Cc1cc(C)cc(C(=O)N(CC(=O)NCc2ccccc2)Cc2ccc(C(=O)Nc3ccccc3N)cc2)c1
CC[C@H]1OC(=O)[C@@H](C)[C@H](O[C@@H]2C[C@](C)(OC)[C@@](O)(CN(C)Cc3ccc(-c4cn(CC(=O)NO)nn4)cc3)[C@@H](C)O2)[C@H](C)[C@H](O[C@H]2O[C@@H](C)C[C@@H](N(C)C)[C@@H]2O)[C@@](C)(O)C[C@H](C)CN(C)[C@@H](C)[C@H](O)[C@@]1(C)ONc1ccccc1NC(=O)CNC(=O)/C(Cc1ccc(O)c(Br)c1)=N/O
Cc1cc(-c2nc3sc4c(c3c(=O)[nH]2)CCN(C)C4)cc(C)c1OCc1ccc(C(=O)Nc2ccccc2N)cc1

CCCNNC(=O)c1ccc(CNC(=O)c2cc3ccccc3cn2)cc1
O=C(NO)c1coc(CCCN2CCC(CN[C@H]3C[C@@H]3c3ccccc3)CC2)n1
CCCC(Oc1cc(N(C)c2nc(C)nc3ccccc23)ccc1OC)C(=O)NO
CN1CCN(C(=O)c2ccc(-c3noc(C(F)(F)F)n3)cc2)CC1
O=C(c1ccc(-c2noc(C(F)(F)F)n2)cc1)N1CCN(c2ccccc2)CC1
CNC(=O)CNC(=O)c1ccc(-c2noc(C(F)(F)F)n2)cc1
CC(=O)N1CCC(NC(=O)c2ccc(-c3noc(C(F)(F)F)n3)cc2)CC1
O=C(Nc1ccnc(Cl)c1)c1ccc(-c2noc(C(F)(F)F)n2)cc1
NC1CCCCC1NC(=O)c1ccc(-c2noc(C(F)(F)F)n2)cc1
COc1ccc(N(C)c2nc(C)nc3ccccc23)cc1OC(C)(C)C(=O)NO
COc1ccc(N(C)c2nc(C)nc3ccccc23)cc1OC(C(=O)NO)c1ccccc1
CCCNNC(=O)c1ccc(CNC(=O)c2cc3cc(Br)ccc3o2)cc1
Nc1ccccc1NC(=O)CCCCCN1C(=O)c2cccc3cccc(c23)C1=O
Nc1ccccc1NC(=O)CCCCCN1C(=O)c2cccc3c(N4CCOCC4)ccc(c23)C1=O
O=C(CNC(=O)/C(Cc1ccc(O)c(Br)c1)=N/O)NO
O=C(CCS)NCCCNC(=O)c1cc(-c2ccc(F)cc2)[nH]n1
O=C(CCS)NCCCCNC(=O)c1cc(-c2ccc(F)cc2)[nH]n1
COc1cc(OCc2cccc(-c3ccccc3)c2C)cc(OC)c1CNCCC(=O)NO
COc1cc2c(NC3CCN(C)CC3)cc(-c3ccc(C)o3)nc2cc1OCCCN1CCCC1
CN(C)C(=O)c1ccc(-c2noc(C(F)(F)F)n2)cc1
CNC(=O)Cc1ccc(-c2noc(C(F)(F)F)n2)cc1
O=C(NC1CCCCC1)c1ccc(-c2noc(C(F)(F)F)n2)cc1
CCN(C)C(=O)c1ccc(-c2noc(C(F)(F)F)n2)cc1
O=C(c1ccc(-c2noc(C(F)(F)F)n2)cc1)N1CCCCC1
CC(C)NC(=O)c1ccc(-c2noc(C(F)(F)F)n2)cc1
O=C(NC1CCCC1)c1ccc(-c2noc(C(F)(F)F)n2)cc1
O=C(c1ccc(-c2noc(C(F)(F)F)n2)cc1)N1CCC(Cc2ccccc2)CC1
CC(CN(C)C)NC(=O)c1ccc(-c2noc(C(F)(F)F)n2)cc1
CCOc1ccc(CN2CC(C(=O)NO)C2)cc1-c1nc2c(CC)nn(C)c2c(=O)[nH]1
CCC(=O)CCCCC[C@@H]1NC(=O)[C@H](CCCCNC(C)=O)NC(=O)C[C@H](CC(C)C)NC(=O)[C@H](Cc2c[nH]c3ccccc23)NC1=O
Nc1ccccc1NC(=O)CCCCCN(CC(=O)NC1CCCCC1)C(=O)c1ccccc1
Cc1cc(C)cc(C(=O)N(CCCCCC(=O)Nc2ccccc2N)CC(=O)NC2CCCCC2)c1
CC(C)c1cc(C(=O)N2Cc3ccc(NC(=O)CCC(=O)NO)cc3C2)c(O)cc1O
CN(C)CC(C)(C)NC(=O)c1ccc(-c2noc(C(F)(F)F)n2)cc1F
CC(C)[C@H](CN(C)C)NC(=O)c1ccc(-c2noc(C(F)(F)F)n2)cc1
CN(C)CCN(C)C(=O)c1ccc(-c2noc(C(F)(F)F)n2)cc1
O=C(NCCc1ccccc1)c1ccc(-c2noc(C(F)(F)F)n2)cc1
O=C(NC1CCNC1)c1ccc(-c2noc(C(F)(F)F)n2)cc1
Nc1cc(F)ccc1NC(=O)c1ccc(CCNc2nc(N)n3nc(-c4ccco4)nc3n2)cc1
COC(=O)CCCCCCCOc1ccc(S(=O)(=O)N2CCOCC2)cc1NC(=O)c1c2c(c(C)n1C)C(=O)CCCC2
Nc1ccccc1NC(=O)/C=C/c1cnc(-c2ccc3cc[nH]c3c2)s1
CCCCNC(=O)/C(Cc1ccc(O)c(Br)c1)=N/O
CCCNNC(=O)c1ccc(CNC(=O)c2cc3cc(OC)ccc3[nH]2)cc1
CN(C)c1ccc(C(=O)N(CC(=O)NCc2ccccc2)Cc2ccc(C(=O)Nc3ccccc3N)cc2)cc1
O=C(NCCO)/C(Cc1ccc(O)c(Br)c1)=N/O
Cc1cc(C)cc(CNC(=O)CN(CCCCCC(=O)Nc2ccccc2N)C(=O)c2cc(C)cc(C)c2)c1
Cc1cc(-c2nc3sc4c(c3c(=O)[nH]2)CCN(C)C4)cc(C)c1OCCCCCC(=O)Nc1ccccc1N
Nc1ccccc1NC(=O)CCCCNC(=O)/C(Cc1ccc(O)c(Br)c1)=N/O
C[C@@H](c1nc2c(cnn2C2CCCC2)c(=O)[nH]1)N1CC(Cc2ccc(C(=O)Nc3ccccc3N)cc2)C1
O=C(NO)c1cccc2c1CN(c1nc3ccccc3[nH]1)C2
CC[C@H]1OC(=O)[C@H](C)[C@@H](O[C@@H]2C[C@](C)(OC)[C@H](O)[C@@H](C)O2)[C@H](C)[C@@H](O[C@@H]2O[C@H](C)C[C@H](N(C)C)[C@H]2O)[C@@](C)(O)C[C@@H](C)CN(Cc2ccc(-c3cn(CC(=O)NO)nn3)cc2)[C@@H](C)[C@@H](O)[C@]1(C)O

CC[C@H]1OC(=O)[C@H](C)C(=O)[C@H](C)[C@@H](O[C@@H]2O[C@H](C)C[C@H](N(C)Cc3ccc(-c4cn(CC(=O)NO)nn4)cc3)[C@H]2O)[C@@](C)(OC)C[C@@H](C)C2=NCCN3C(=O)O[C@@]1(C)[C@H]3[C@H]2C
CCCNNC(=O)c1ccc(CNC(=O)c2cc3cc(Cl)ccc3o2)cc1
CCCNNC(=O)c1ccc(CNC(=O)c2ccc3ccncc3c2)cc1
Nc1ccccc1NC(=O)c1ccc(CCCNc2nc(N)n3nc(-c4ccco4)nc3n2)cc1
Nc1ccccc1NC(=O)c1ccc(CNc2nc(N)n3nc(-c4ccco4)nc3n2)cc1
Cc1ccc(-c2ncc(/C=C/C(=O)Nc3ccccc3N)s2)cc1
C/C=C1\NC(=O)c2csc(n2)CNC(=O)C[C@@H](/C=C/CCSSCCCCCCC)OC(=O)[C@H](CCCCN)NC1=O
O=C(O)c1ccc(CCCN2CCC(CN[C@H]3C[C@@H]3c3ccccc3)CC2)cc1
Nc1ccccc1NC(=O)c1ccc(CCn2ncc3c2nc(N)n2nc(-c4ccco4)nc32)cc1
CCCNNC(=O)c1ccc(CNC(=O)c2cc3ccccc3o2)cc1
COc1ccc(N(C)c2nc(C)nc3ccccc23)cc1OC(C)C(=O)NO
COc1ccc(N(C)c2nc(C)nc3ccccc23)cc1OC1(C(=O)NO)CCC1
CCCCC(Oc1cc(N(C)c2nc(C)nc3ccccc23)ccc1OC)C(=O)NO
COc1ccc(N(C)c2nc(C)nc3ccccc23)cc1OC(C(=O)NO)C(C)C
CCCNNC(=O)c1ccc(CNC(=O)c2nc3ccccc3[nH]2)cc1
CCCNNC(=O)c1ccc(CNC(=O)c2ccc3cnccc3c2)cc1
Nc1nc(-c2ccco2)c2cnn(CCc3ccc(C(=O)Nc4ccccc4N)cc3)c2n1

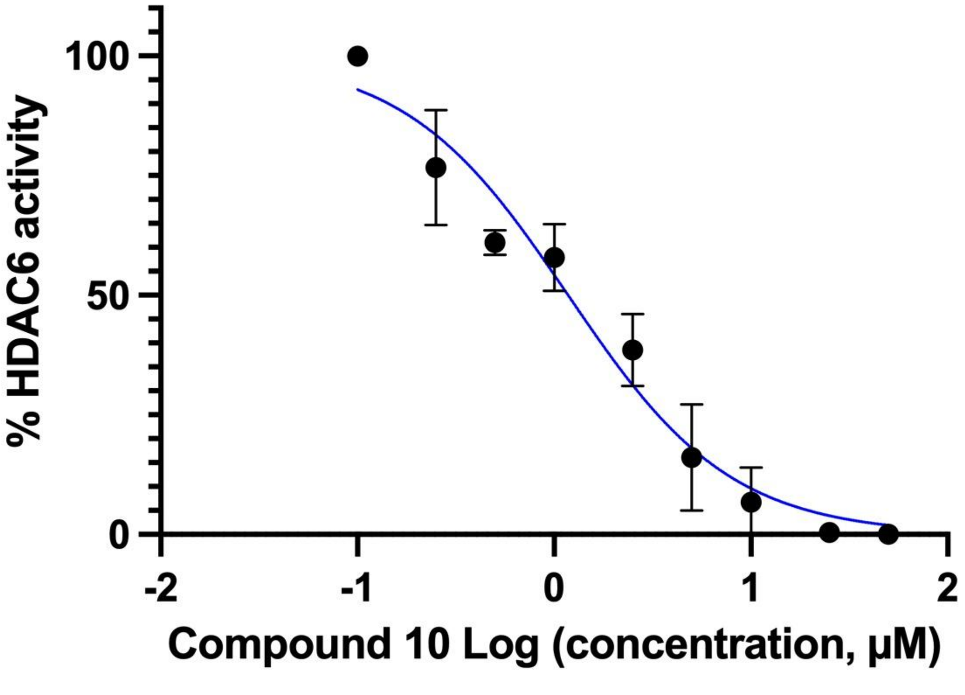

Figure S5. Dose-response curve analysis of HDAC6 activity in the presence of increasing concentrations of Compound 10. Enzymatic activity is expressed as a percentage of residual activity relative to control (DMSO).

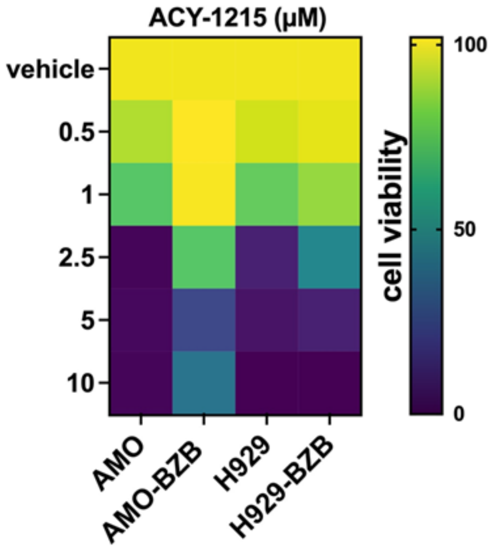

MM cell lines	IC_{50} ACY-1256
AMO	1.55 μM ± 0.12
AMO-BZB	2.38 μM ± 0.07
H929	1.45 μM ± 0.11
H929-BZB	4.17 μM ± 0.67

Figure S6. Heatmap showing cell viability assessed by the CellTiter-Glo assay in MM cell lines treated with ACY-1215 or vehicle control (DMSO) for 72 hours. Viability is expressed as a percentage relative to vehicle-treated cells. The half-maximal inhibitory concentrations (IC_{50}) of ACY-1215 for AMO, AMO-BZB, H929, and H929-BZB cell lines are reported in the accompanying table. IC_{50} values (mean ± SD) were calculated using GraphPad Prism software based on three independent experiments.

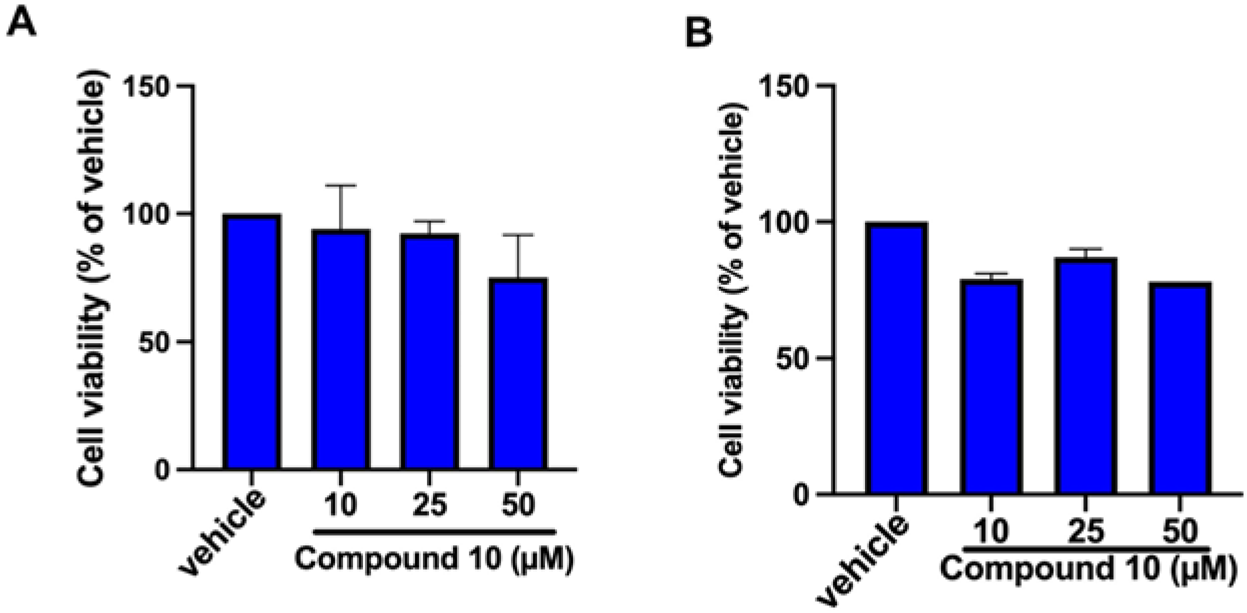

Figure S7. Cell viability assessed by the CellTiter-Glo assay in 293T (**A**) or healthy PBMCs (**B**), 72 hours after treatment with Compound 10. Viability is expressed as a percentage relative to vehicle-treated cells.

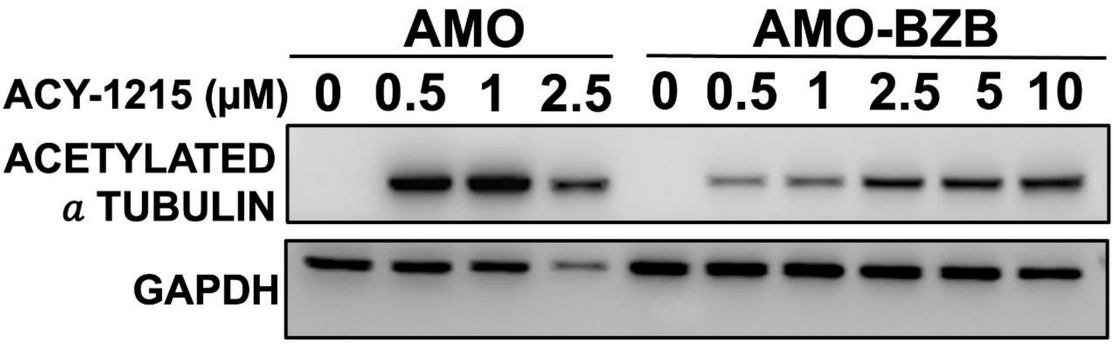

Figure S8. Western blot analysis of acetylated α-tubulin in AMO and AMO-BZB cells, 48 hours after treatment with ACY-1215. GAPDH was used as a loading control.

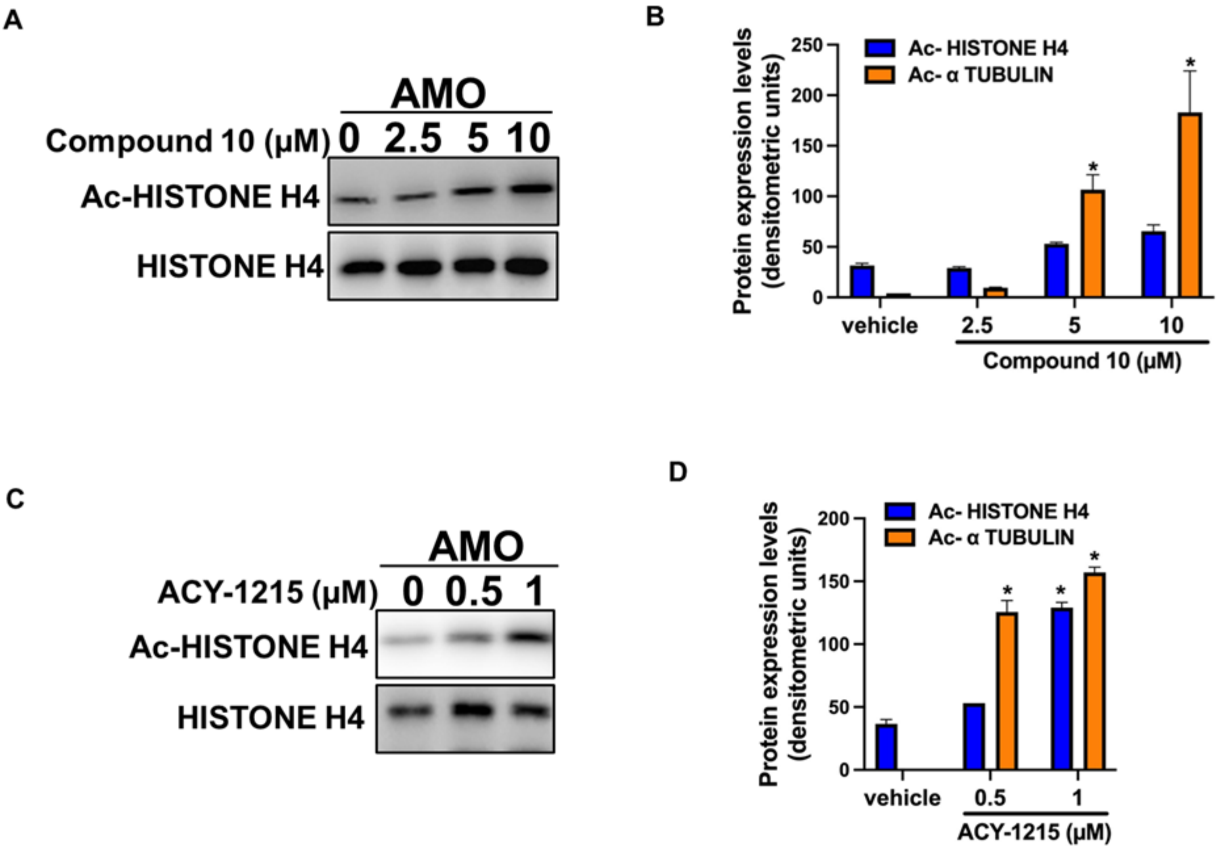

Figure S9. **Western Blot Analysis of Histone H4 and α-Tubulin Acetylation in AMO Cells Treated with Compound 10 and ACY1215. A**) Western blot analysis of acetylated- H4 and histone H4 in AMO cells treated for 72 hours with Compound 10. **B**) Normalized expression of acetylated-H4 and acetylated- α-tubulin in AMO cells, 72 hours after treatment with Compounds 10. **C**) Western blot analysis of acetylated- H4 and histone H4 in AMO cells treated for 48 hours with ACY1215. **D**) Normalized expression of acetylated-H4 and acetylated- α-tubulin in AMO cells, 48 hours after treatment with ACY-1215. GAPDH was used a loading control. *$p<0.05$ compared to corresponding vehicle.

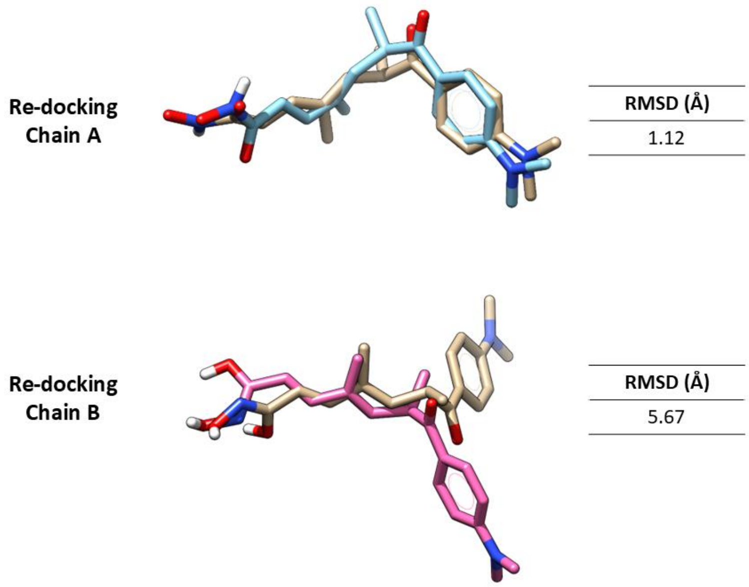

Figure S10. Three-dimensional visualization of redocking analysis. Superimposition of the docked ligand pose with the crystallographic conformation of the reference ligand within the HDAC6 active site. For chain A and chain B, the docked ligand is shown in light blue and pink, respectively.
